# A mitochondria-to-nucleus regulation mediated by the nuclear-translocated mitochondrial lncRNAs

**DOI:** 10.1371/journal.pgen.1011580

**Published:** 2025-01-27

**Authors:** Jia Li, Ruoling Bai, Yulian Zhou, Xu Song, Ling Li

**Affiliations:** Center for Functional Genomics and Bioinformatics, Key Laboratory of Bio-Resource and Eco-Environment of Ministry of Education, College of Life Sciences, Sichuan University, Chengdu, Sichuan, China; Universita degli Studi di Bari Aldo Moro, ITALY

## Abstract

A bidirectional nucleus-mitochondria communication is essential for homeostasis and stress. By acting as critical molecules, the nuclear-encoded lncRNAs (nulncRNAs) have been implicated in the nucleus-to-mitochondria anterograde regulation. However, role of mitochondrial-derived lncRNAs (mtlncRNAs) in the mitochondria-to-nucleus retrograde regulation remains elusive. Here, we identify functional implication of the mtlncRNAs MDL1AS, lncND5 and lncCyt b in retrograde regulation. Mediated by HuR and PNPT1 proteins, the mtlncRNAs undergo a mitochondria-to-nucleus traveling and then regulate a network of nuclear genes. Moreover, as an example of the functional consequence, we showed that the nuclear-translocated lncCyt b cooperates with the splicing factor hnRNPA2B1 to influence several aspects of cell metabolism including glycolysis, possibly through their regulatory effect on the post-transcriptional processing of related nuclear genes. This study advances our knowledge in mitochondrial biology and provides new insights into the role of mtlncRNAs in mitochondria-nucleus communications.

## Author summary

Due to the essential role of the nucleus-mitochondria crosstalk in homeostasis and stress, identifying and characterizing critical molecules that contribute to the nucleus-to-mitochondria (anterograde) and mitochondria-to-nucleus (retrograde) communication is fundamental to human biology. In mammals, mitochondria are the only organelles that possess their own genetic material (mtDNA), which produces a set of lncRNAs (mitochondrial-derived lncRNAs, mtlncRNAs). Many lncRNAs encoded by nuclear genome (nuclear-encoded lncRNAs, nulncRNAs) have been involved in the regulation of mitochondrial function. Nevertheless, whether and how the mtlncRNAs operate in the retrograde regulation signaling to modulate nuclear events remains largely unclear. Here, we identify that the mtlncRNAs MDL1AS, lncND5 and lncCyt b undergo a mitochondria-to-nucleus traveling and then regulate a network of nuclear genes, and describe an example of how the nuclear-translocated lncCyt b modulates nuclear genes at the post-transcriptional level via its binding partner, the splicing factor hnRNPA2B1, to influence several aspects of cell metabolism including glycolysis, thereby acting as a critical regulatory molecule in retrograde signaling. Our study illustrates that certain mtlncRNAs, on the basis of their nuclear translocation, can operate in the mitochondria-to-nucleus retrograde regulation, advancing knowledge in mitochondrial biology as well as our understanding of implication of mtlncRNAs in the regulation of inter-compartmental crosstalk in cells.

## Introduction

Mitochondria are the only organelles in mammalian cells that possess their own genetic material (mitochondrial DNA, mtDNA) and gene expression machineries [1, 2]. Nevertheless, they are still considered as semi-autonomous entities because most of mitochondrial proteins are encoded by the nuclear genes and most mitochondrial activities are run in a nucleus-relying manner [3, 4]. In addition to their role in energy generation, mitochondria also act as stress sensors and integrators to modulate nuclear gene expression, thereby playing crucial roles in a wide range of essential biological processes such as cell division, differentiation and cell death [5, 6]. It is becoming increasingly clear that a bidirectional communication between nucleus and mitochondria is required for the maintenance of whole cell homeostasis and the adaptation to cellular stress [7, 8]. Thus, identifying critical molecules that facilitate the nucleus-to-mitochondria (anterograde) or mitochondria-to-nucleus (retrograde) communication, as well as elucidating their mechanisms of action in this inter-compartmental crosstalk, is fundamental to human biology.

In mammals, the vast majority of nuclear genome is ubiquitously transcribed to generate long noncoding RNAs (lncRNAs). A sizable fraction of nuclear-encoded lncRNAs (nulncRNAs) function in cells by regulating gene expression at multiple levels [9]. Moreover, specific nulncRNAs were demonstrated to regulate mitochondrial network by triggering an anterograde signaling [10, 11]. Intriguingly, current studies also identified a set of mtDNA-generated lncRNAs (mtlncRNAs) [12, 13]. Given the functional implication of nulncRNAs in anterograde regulation, it is an interesting issue to determine whether and how the mtlncRNAs operate in the retrograde regulation signaling to influence nuclear events. Our current data reveal that several mtlncRNAs undergo a mitochondria-to-nucleus traveling, in which the RNA binding proteins HuR and PNPT1 play critical roles, and show an example of the underlying biological significance that the mtlncRNA lncCyt b, following its nuclear translocation, cooperates with the splicing factor hnRNPA2B1 to modulate pre-mRNA processing and mRNA maturation of specific genes for the regulation of cell metabolism such as glycolysis.

## Results

### Several mtlncRNAs undergo a mitochondria-to-nucleus translocation

**Fig 1:**
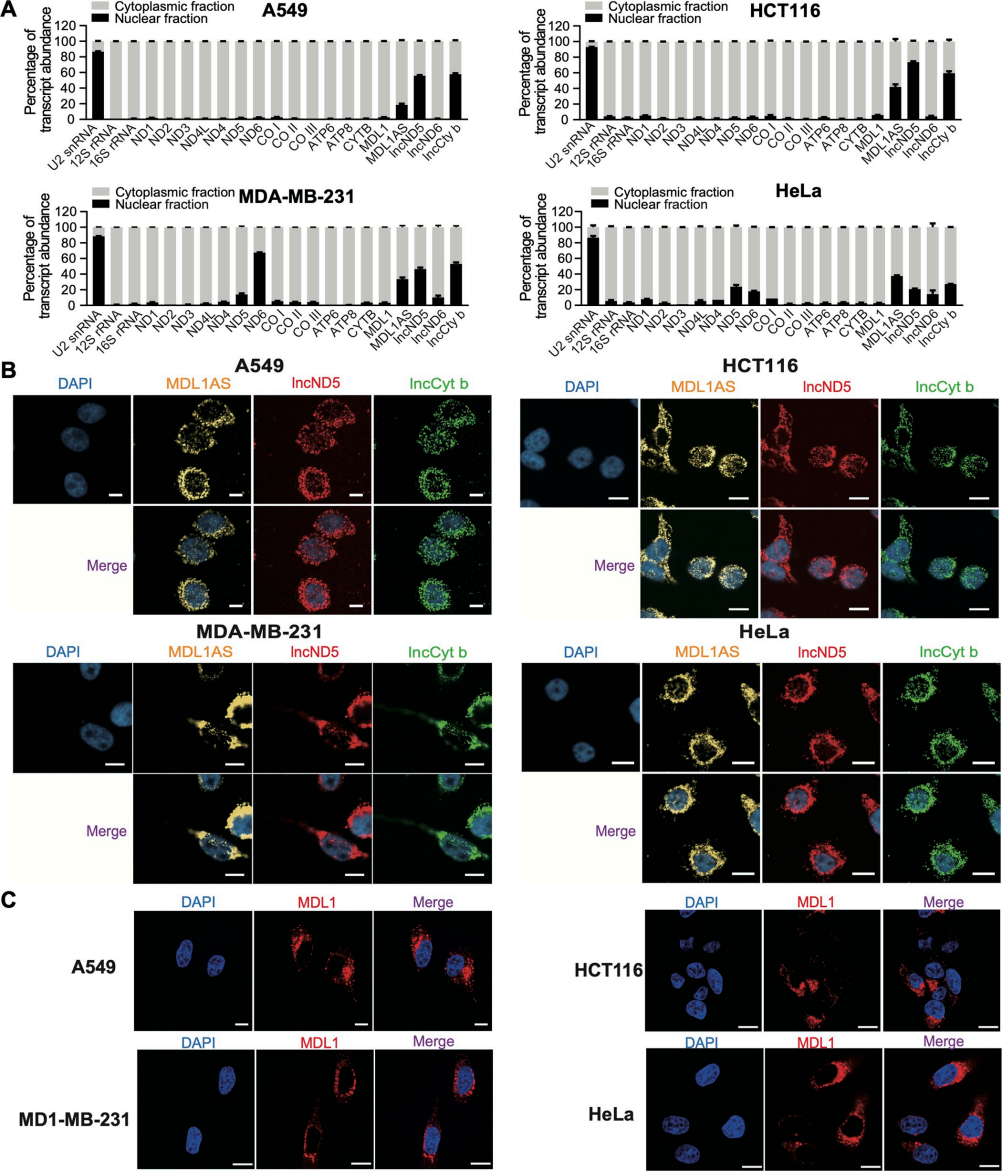
Several mtlncRNAs undergo a mitochondria-to-nucleus translocation. (A) RT-qPCR assay following nuclear/cytoplasmic fractionation detecting the distribution of the indicated mitochondrial RNAs in a panel of tumor cells. U2 snRNA, a canonical nuclear-retained transcript, and the mitochondrial 12S and 16S rRNAs, were assessed as controls to confirm the findings of our nuclear/cytoplasmic fractionation. Data are shown as means ± standard deviation (SD) of n = 3 independent experiments. (B,C) RNA FISH detecting the distribution of MDL1AS, lncND5 and lncCyt b (B), as well as the distribution of MDL1 (C), in a panel of cell lines. Scale bars, 10 μm.

The finding that many nuclear-encoding noncoding transcripts, such as 5S rRNA and tRNAs [14], reside also in mitochondria prompted an inverse search for the mtlncRNAs that can be transported into the nucleus. Five mtlncRNAs, along with a set of mitochondrial-generated rRNAs and mRNAs (S1A Fig), were analyzed following cellular fractionation for their localization in a panel of tumor and normal cells. Three mtlncRNAs, including MDL1AS, lncND5 and lncCyt b, were revealed to exhibit a distribution pattern similar to the nuclear-localized U2 snRNA in all tested cells; their distribution, however, is clearly distinct from that of the other mitochondrial transcripts, except for the ND6 mRNA in MDA-MB-231 cells (Figs 1A; S1B and S1C). RNA fluorescence in situ hybridization (RNA FISH) also showed that the three mtlncRNAs have a nuclear distribution (Figs 1B, S2A). As our previous report [15], the mtlncRNA MDL1 exhibits an extranuclear localization pattern (Figs 1A, 1C, S1B). Since mtDNA is capable of encoding circular RNAs (mecciRNAs) [16], we explored whether the three mtlncRNAs are also produced as mecciRNAs. Our results showed that all three mtlncRNAs were sensitive to RNases R treatment, indicating that they are linear rather than circular transcripts (Fig S2B). We also assessed their abundance in A549 cells, and the results not only confirmed their simultaneous distribution in nucleus and cytoplasm but also revealed that they present in high copies in cells (Fig S2C). Thus, our results uncover a mitochondria-to-nucleus translocation for the mtlncRNAs MDL1AS, lncND5 and lncCyt b, and suggest that their nuclear translocation should be a general event occurring in multiple cellular contexts and that, depending on their nuclear translocation, the three mtlncRNAs may operate in the mitochondria-to-nucleus retrograde regulation.

### HuR facilitates nuclear translocation of mtlncRNAs

**Fig 2:**
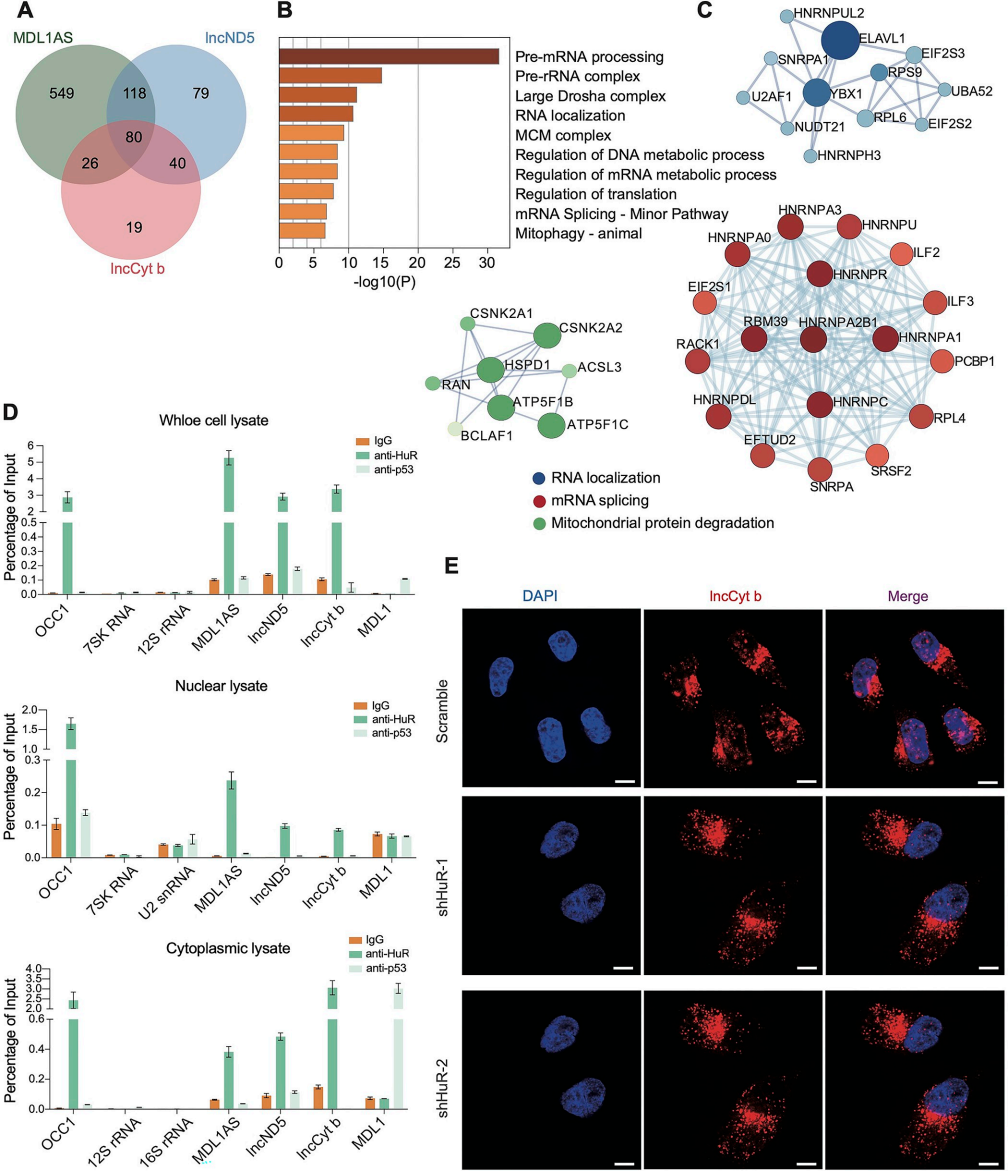
HuR facilitates nuclear translocation of mtlncRNAs. (A) Venn diagram showing overlap of unique proteins bound by MDL1AS, lncND5 and lncCyt b in A549 cells. (B,C) GO enrichment (B) and STRING network (C) analyses of the 80 common proteins bound by MDL1AS, lncND5 and lncCyt b. (D) Native RIP followed by RT-qPCR detecting the indicated RNA transcripts retrieved by anti-HuR antibody or by normal IgG and anti-p53 antibody in whole-cell, nuclear or cytoplasmic lysates of A549 cells. The HuR-binding lncRNA OCC1 and p53-binding mtlncRNA MDL1 was included as controls [15]. The retrieved RNAs are presented as a percentage of the amount input. Data are shown as means ± SD of n = 3 independent experiments. (E) RNA FISH detecting the distribution of lncCyt b in control and HuR-depleted A549 cells. Scale bars, 10 μm.

We set out to dissect the mechanism underlying nuclear translocation of the mtlncRNAs by identifying their putative protein partners. We incubated biotinylated MDL1AS, lncND5 or lncCyt b, with their antisense counterparts being included as controls, with lysates of A549 cells, and employed mass spectrometry to identify the proteins co-precipitated with RNA baits (S3A, S1 Table). The above procedures isolated 80 common protein partners of the three mtlncRNAs, and these proteins were found to be substantially enriched in pre-mRNA processing, RNA localization, and RNA metabolism by Gene Ontology (GO) and Protein-Protein Interaction (PPI) analyses (Fig Fig 2A–2C). Among the 80 common protein partners, HuR, also known as ELAVL1, was attractive and subjected to subsequent investigation because of its well-established implication in the control of RNA metabolism and RNA localization [17–20]. Western blot following RNA pull-down verified the reciprocal interaction between HuR and the mtlncRNAs (S3B Fig). Native ribonucleoprotein immunoprecipitation (RIP) further showed that the HuR antibody, relative to IgG control, retrieved a substantial amount of the three mtlncRNAs in A549 cells, and that the HuR interaction with the mtlncRNAs could be detected in both nucleus and cytoplasm (Fig 2D). By serving as a carrier protein, HuR was reported to promote the nucleocytoplasmic trafficking of both protein-coding and noncoding RNA transcripts [17–20]. Here, we further determined whether HuR has the ability to facilitate the nuclear translocation of MDL1AS, lncND5 and lncCyt b. HuR knockdown was mediated by two independent short hairpin RNAs (shRNAs) (S3C Fig). Concomitantly, we observed that HuR knockdown resulted in a decreased nuclear distribution of the mtlncRNAs (Figs 2E and S3D). Given the regulatory effect of HuR on RNA stability, we tested whether HuR knockdown affects stability of the three mtlncRNAs. The stability of Fos mRNA, which was included as an experimental control [21], was decreased in the case of HuR knockdown, but the stability of the three mtlncRNAs was not changed under the same condition. (S3E Fig).

### HuR facilitates mtlncRNA nuclear translocation via its binding to particular RNA regions

**Fig 3:**
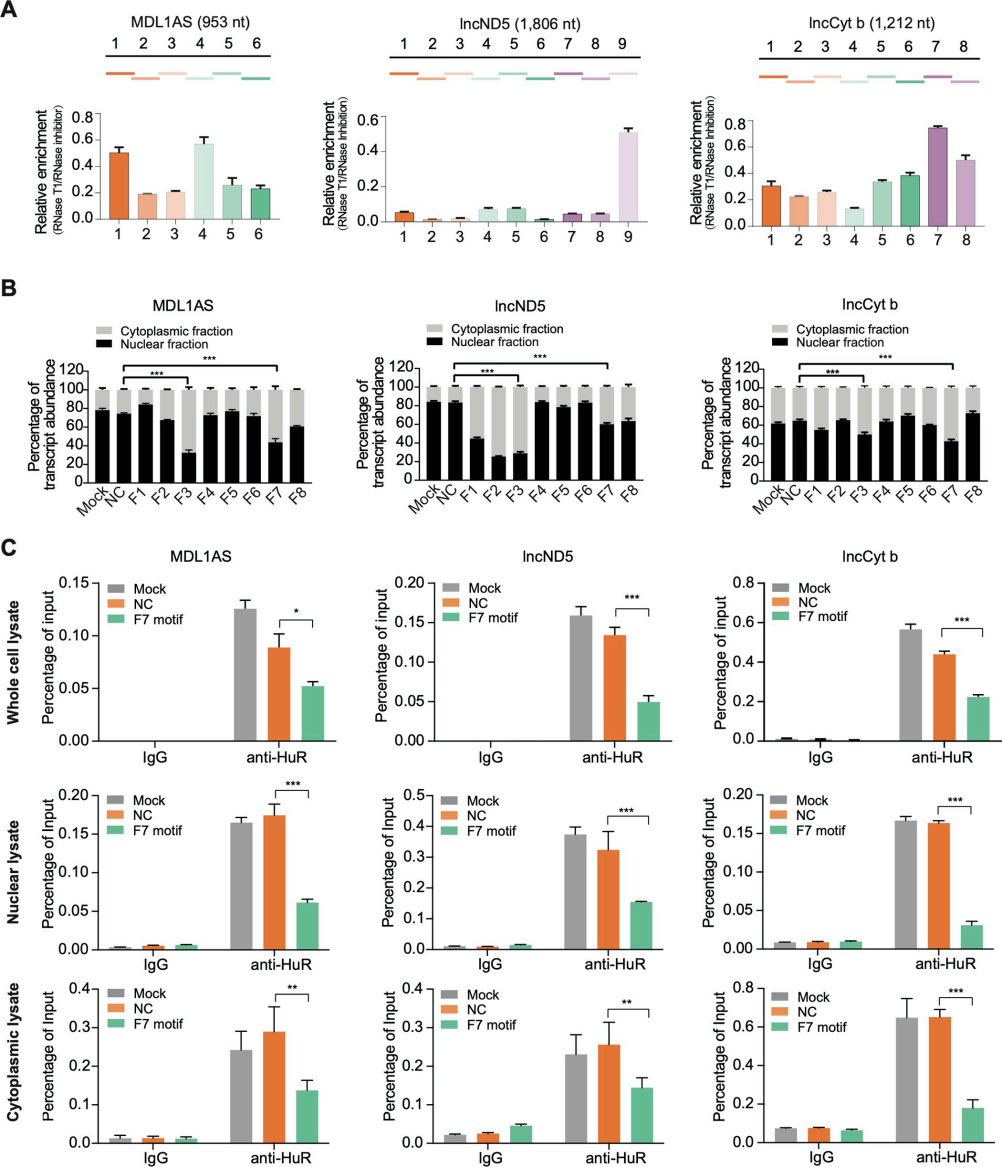
HuR facilitates nuclear translocation of mtlncRNAs via its binding to specific mtlncRNA regions. (A) RIP-based mapping assay detecting the association of HuR protein with different regions within MDL1AS, lncND5 and lncCyt b, which are illustrated on the top. Results are presented relative to the RNase inhibitor control. Data are shown as means ± SD of n = 3 independent experiments. (B) Nuclear/cytoplasmic fractionation followed by RT-qPCR detecting the influence of the overexpressed lncCyt b segments on nuclear distribution of MDL1AS, lncND5 and lncCyt b in A549 cells. A 16S rRNA segment was overexpressed as a negative control (NC). Data are shown as means ± SD of n = 3 independent experiments. ***P < 0.001 by Student’s *t* test. (C) Native RIP followed by RT-qPCR detecting effect of the overexpressed HuR-binding lncCyt b segment F7 on HuR association with the endogenous MDL1AS, lncND5 and lncCyt b in whole-cell, nuclear or cytoplasmic lysates of A549 cells. A 16S rRNA segment was overexpressed as a negative control (NC). The retrieved RNAs are presented as a percentage of the amount input. Data are shown as means ± SD of n = 3 independent experiments. *P < 0.05, **P < 0.01, ***P < 0.001 by Student’s *t* test.

Subsequently, we sought to elucidate the molecular basis whereby HuR functions in nuclear translocation of the mtlncRNAs. For this purpose, we first set up a RIP-based mapping assay to identify the functional regions of the mtlncRNAs that are responsible for their HuR binding. Nuclear extracts from A549 cells were subjected to a limited RNase T1 digestion, so that the mtlncRNA regions protected by the bound HuR would remain uncleaved. After HuR RIP, the enriched RNA fragments were identified by RT-qPCR analysis using primer sets that cover the entire mtlncRNA sequence in overlapping ~170 nt-long segments. Our results showed that several regions within the mtlncRNAs were enriched by HuR antibody after RNase T1 treatment (3A Fig), hinting their involvement in HuR binding. The mtlncRNA segments identified by RIP-based mapping assay were also subjected to enzyme-linked immunosorbent assay (ELISA) for confirmation of their association with HuR protein (S4A Fig). Next, we investigated whether the HuR-binding region is essential for the nuclear translocation of the mtlncRNAs. Specially, the procedure involved overexpressing a serial of lncCyt b segments, including the identified HuR-binding segment F7, in cells and analyzing the effect of each lncCyt b segment on nuclear translocation of the endogenous mtlncRNAs. As revealed, the overexpressed lncCyt b segment F7 has the ability to decrease nuclear distribution of the endogenous MDL1AS, lncND5 and lncCyt b (Figs 3B; S4B, S4C). Mechanistically, the lncCyt b segment F7, by native RIP and competitive ELISA assays, was found to serve as a competitor to weaken the reciprocal interaction between HuR and the nuclear-translocated mtlncRNAs (Figs 3C, S4D), and the competitive effect appears to occur in both cytoplasm and nucleus (Fig 3C). Taken together, the results suggest that, in addition to its implication in nucleocytoplasmic trafficking of nuclear-encoded RNAs, HuR also acts in a contrary direction to facilitate nuclear translocation of specific mtlncRNAs.

### PNPT1 is another protein contributing to the nuclear translocation of mtlncRNAs

In addition to the lncCyt b segment F7, several lncCyt b segments outside the HuR-binding region, such as the lncCyt b segment F3, were found to also decrease the nuclear distribution of MDL1AS, lncND5 and lncCyt b (Fig 3B), hinting that certain factor other than HuR, possibly depending on its association with mtlncRNAs, may also contribute to nuclear translocation of the three mtlncRNAs.

**Fig 4:**
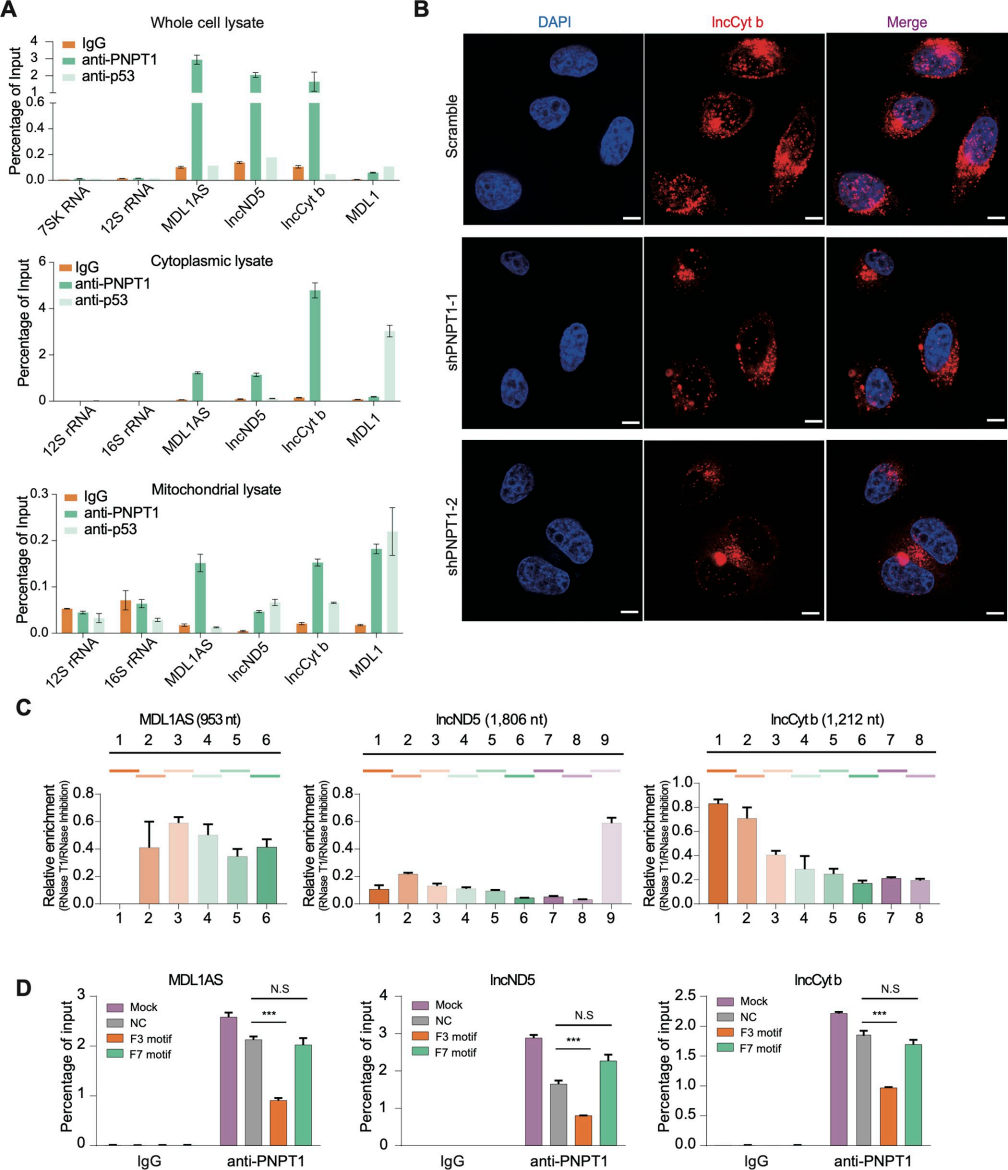
PNPT1 contributes to the nuclear translocation of mtlncRNAs. (A) Native RIP followed by RT-qPCR detecting the indicated RNA transcripts retrieved by anti-PNPT1 antibody or by normal IgG and anti-p53 antibody in whole-cell, nuclear or cytoplasmic lysates of A549 cells. The retrieved RNAs are presented as a percentage of the amount input. Data are shown as means ± SD of n = 3 independent experiments. (B) RNA FISH detecting the distribution of the indicated mtlncRNAs in control and PNPT1-depleted A549 cells. Scale bars, 10 μm. (C) RIP-based mapping assay detecting the association of PNPT1 protein with different regions within MDL1AS, lncND5 and lncCyt b, which are illustrated on the top. Results are presented relative to the RNase inhibitor control. Data are shown as means ± SD of n = 3 independent experiments. (D) Native RIP followed by RT-qPCR detecting the effect of the overexpressed PNPT1-binding lncCyt b segment (F3) and HuR-binding lncCyt b segment (F7) on PNPT1 association with the endogenous MDL1AS, lncND5 and lncCyt b in cytoplasmic lysates of A549 cells. A 16S rRNA segment was overexpressed as a negative control (NC). The retrieved RNAs are presented as a percentage of the amount input. Data are shown as means ± SD of n = 3 independent experiments. ***P < 0.001 by Student’s *t* test. N.S, not significant.

Polyribonucleotide nucleotidyltranserase 1 (PNPT1) is a multifunctional protein that plays critical roles in mitochondrial homeostasis [22, 23]. PNPT1 is distributed both in cytoplasm and the mitochondria [22, 23], and the PNPT1 localized in mitochondrial intermembrane space (MIS) was reported to act as a “carrier” that mediates the mitochondrial import of different types of nuclear-encoded RNAs [24]. Here, we sought to assess its potential involvement in the nuclear translocation of the three mtlncRNAs. By native RIP and RNA pull-down assays, PNPT1 was identified to associate with MDL1AS, lncND5 and lncCyt b (Figs 4A and S5A). Importantly, results from cellular fractionation and RNA FISH experiments showed that PNPT1 knockdown decreased nuclear distribution of the mtlncRNAs (Figs 4B, S5B and S5C). On the other hand, RIP-based mapping and ELISA assays identified several mtlncRNA regions, including those localized at the 5’ end of lncCyt b, as the major PNPT1-binding domains (Figs 4C and S5D); moreover, native RIP and competitive ELISA experiments confirmed the PNPT1-binding lncCyt b segment F3 to be a competitor that attenuated the reciprocal interaction between PNPT1 and the nuclear-translocated mtlncRNAs (Figs 4D and S5E). The results therefore may provide a plausible molecular explanation for the aforementioned finding that, in addition to the HuR-binding lncCyt b segment F7, the lncCyt b segment F3 also has an ability to decrease the nuclear distribution of MDL1AS, lncND5 and lncCyt b (Fig 3B). Despite the finding that both HuR and PNPT1 interact with the nuclear-translocated mtlncRNAs, our co-IP assay barely detected a direct association between them (S6A Fig). Thus, the data presented here appear to uncover a new role for PNPT1 in regulating the translocation of MDL1AS, lncND5 and lncCyt b from mitochondria to cytoplasm.

Poly(A) tail is a well-established element required for nucleocytoplasmic trafficking of nuclear-encoded RNAs [25]. Despite the role of PNPT1 in modulating polyadenylation of mitochondrial RNAs [26], the possibility that poly(A) tail contributes to the PNPT1-mediated mitochondrial export of the mtlncRNAs could be ruled out because knockdown of mtPAP, another enzyme involved in the poly(A) synthesis in mitochondria [27], hardly altered the subcellular distribution of the three mtlncRNAs (S6B–S6D Fig). Taken together, our study identifies both PNPT1 and HuR as protein partners of the nuclear-translocated mtlncRNAs, and implies that they might operate in the mitochondrial export and nuclear import of the mtlncRNAs, respectively, thereby facilitating the whole process of mitochondria-to-nucleus traveling.

### The nuclear-translocated mtlncRNAs modulate a network of nuclear genes

Given the identified nuclear translocation of the mtlncRNAs MDL1AS, lncND5 and lncCyt b, we investigated whether they have the ability to influence expression of nuclear genes after their nuclear translocation, thereby operating in the mitochondria-to-nucleus retrograde regulation. We knocked down the mtlncRNAs in the nucleus of A549 cells with two independent ASOs (S7 Fig), and then profiled the nuclear gene expression by RNA deep sequencing (RNA-seq) (S8A Fig). The nuclear genes regulated by MDL1AS, lncND5 and lncCyt b were subjected to GO analysis, and the results showed that the mtlncRNAs were involved in many essential cellular processes such as nuclear division, endoplasmic reticulum stress and macroautophagy (S8B Fig). Several nuclear genes with specialized biological function were subjected to RT-qPCR validation for their regulation by the mtlncRNAs (S8C Fig).

### lncCyt b cooperates with hnRNPA2B1 to modulate cell metabolism

**Fig 5:**
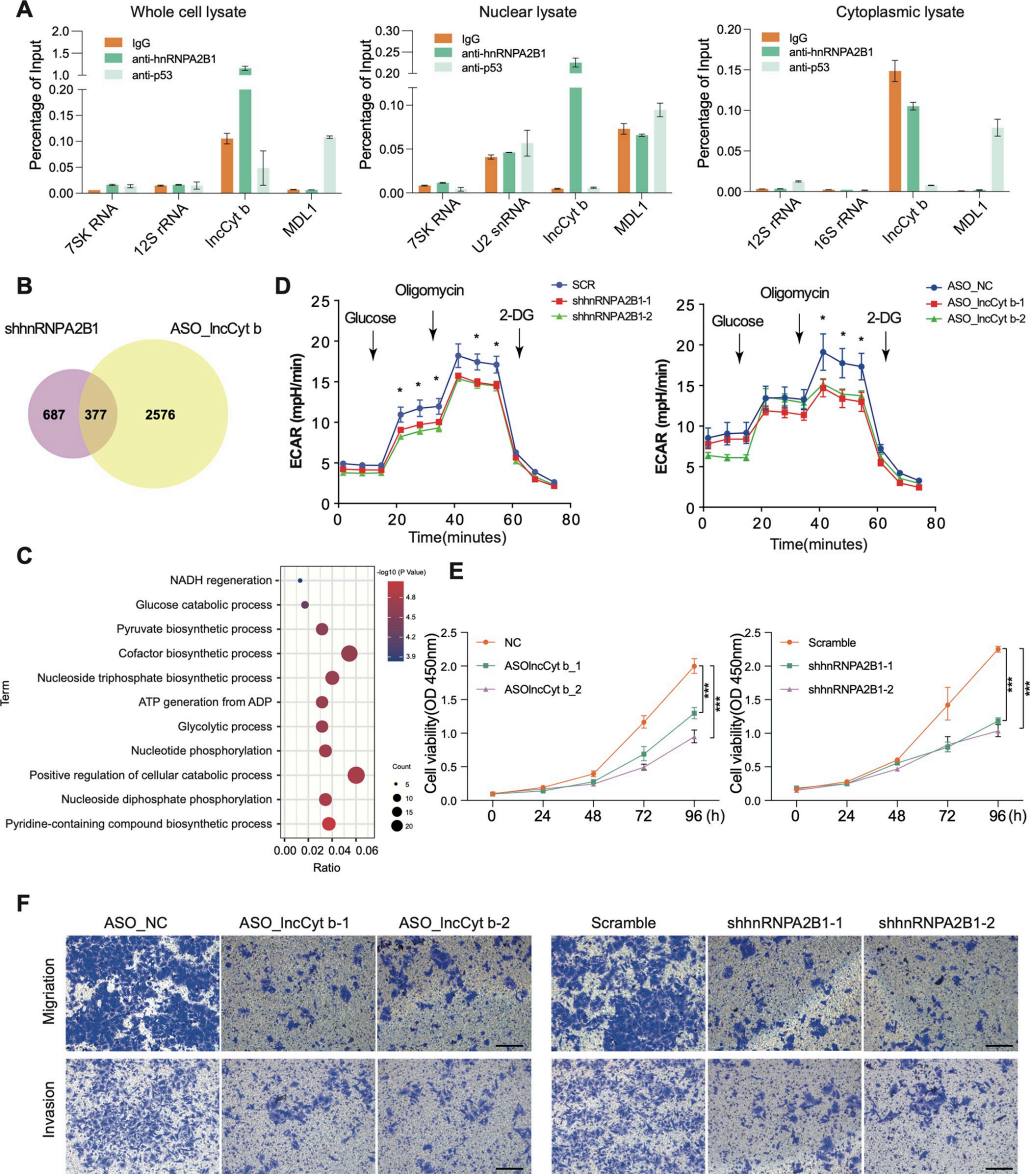
lncCyt b cooperates with hnRNPA2B1 to modulate glycolysis and exhibits physiological significance. (A) Native RIP followed by RT-qPCR detecting MDL1AS, lncND5 and lncCyt b retrieved by anti-hnRNPA2B1 antibody, or by normal IgG and anti-P53 antibody, in whole-cell, nuclear, or cytoplasmic lysates of A549 cells. The retrieved RNAs are presented as a percentage of the amount input. Data are shown as means ± SD of n = 3 independent experiments. (B) Venn diagram showing overlap of nuclear genes regulated by hnRNPA2B1 and lncCyt b in A549 cells. (C) GO enrichment analysis of the nuclear genes co-regulated by hnRNPA2B1 and lncCyt b. (D) Glycolytic stress test showing the reduced glycolytic capacity of A549 cells by hnRNPA2B1 or lncCyt b knockdown. Data are shown as means ± SD of n = 3 independent experiments. *P < 0.05 by Student’s *t* test. (E) CCK-8 assays showing the decreased proliferation of A549 cells by lncCyt b or hnRNPA2B1 knockdown. Data are shown as means ± SD of n = 3 independent experiments. ***P < 0.001 by Student’s *t* test. (F) Transwell assays showing the weakened migration and invasion of A549 cells by lncCyt b or hnRNPA2B1 knockdown. Scale bars, 100 μm.

Next, we took lncCyt b as an example to elucidate potential biological significance of the nucleus-translocated mtlncRNAs. As shown in Fig 2B and 2C, pre-mRNA processing was one of the main pathways enriched by the identified protein partners of lncCyt b, indicating that regulation of pre-mRNA processing may contribute to the functional consequence of lncCyt b following its nuclear translocation. The splicing factor hnRNPA2B1 has a relatively high enrichment score (Fig 2C), and the reciprocal interaction between lncCyt b and hnRNPA2B1 was corroborated by native RIP assay (Fig 5A). Treatment of A549 cells with lncCyt b-targeting ASOs resulted in a decreased level of hnRNPA2B1 protein, with hnRNPA2B1 mRNA level being unaffected (S9A and S9B Fig), suggesting that lncCyt b stabilized hnRNPA2B1 protein after their reciprocal interaction. Furthermore, the results from treatment of control or lncCyt b-depleted A549 cells with MG132 and chloroquine (CQ) indicated that lncCyt b may enhance hnRNPA2B1 stability by inhibiting the proteasomal and lysosomal proteolysis (S9C Fig). Given the effect of lncCyt b on nuclear gene expression (S8 Fig), we proceeded to investigate whether lncCyt b cooperates with hnRNPA2B1 to regulate nuclear genes. RNA-seq identified 377 nuclear genes co-regulated by lncCyt b and hnRNPA2B1 in A549 cells (5B Fig). GO analysis revealed involvement of the co-regulated nuclear genes in many cell metabolism processes including glycolysis (Fig 5C), and the following glycolysis stress test indeed corroborated that the maximum glycolytic stress of A549 cells was reduced by lncCyt b or hnRNPA2B1 knockdown (Fig 5D). Moreover, lncCyt b, as well as its protein partner hnRNPA2B, should have physiological significance because all the tumor lines that we tested exhibited a decreased level of malignant properties, such as proliferation, migration and invasion, upon lncCyt b or hnRNPA2B1 was knocked down (Figs 5E and 5F and S10).

### lncCyt b regulates pre-mRNA processing via the associated hnRNPA2B1

**Fig 6:**
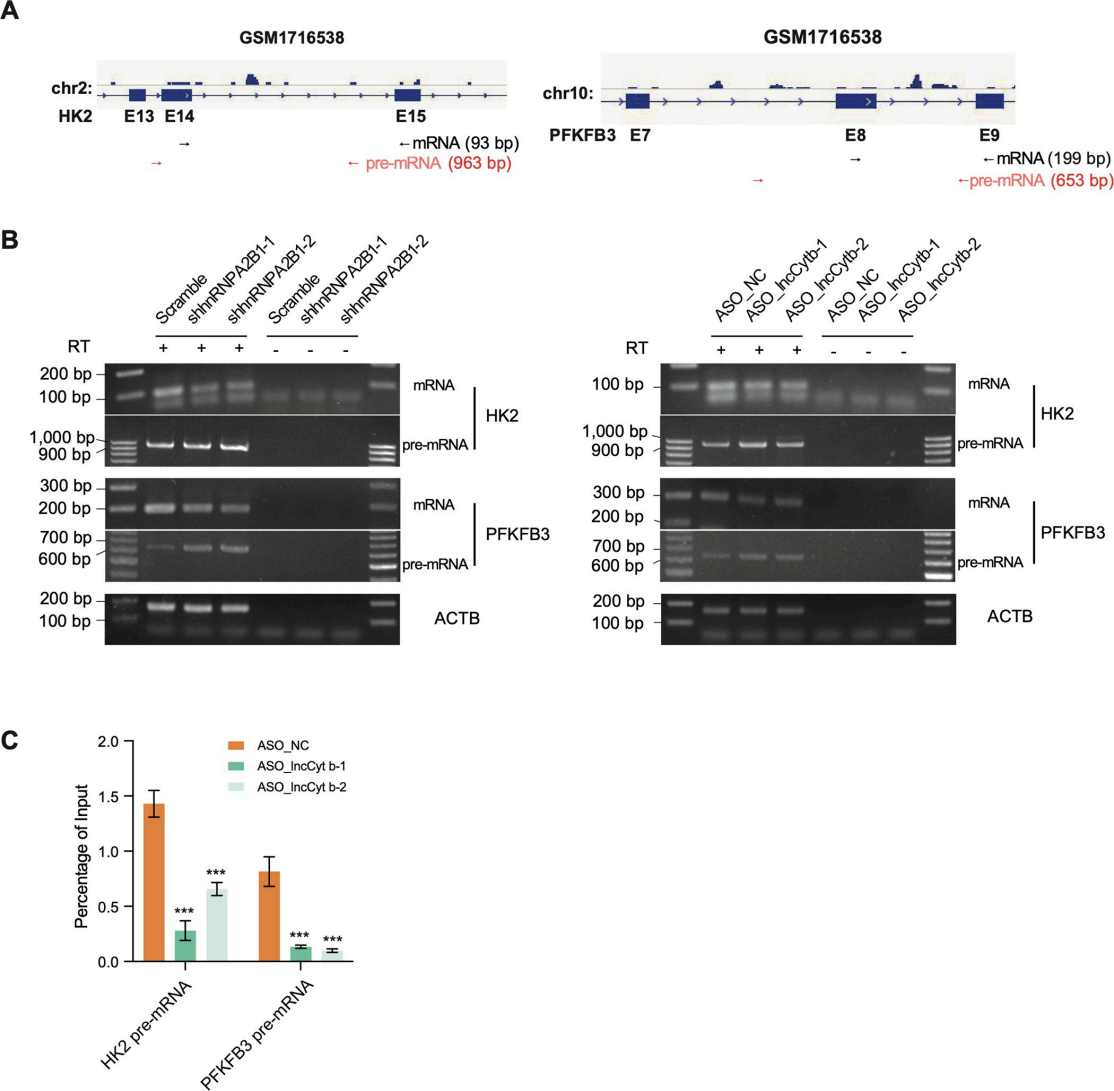
lncCyt b cooperates with hnRNPA2B1 to modulate glycolysis-related nuclear genes. (A) Schematic illustrating density of HITS-CLIP-seq tags for hnRNPA2B1 within the regions covering *HK2* exons 13–15 and *PFKFB3* exons 7–9 (GSM1716538). Primers used for the RT-PCR detection of pre-mRNAs and mature mRNAs, as shown in (B), are illustrated. (B) RT-PCR detecting the influence of hnRNPA2B1or lncCyt b knockdown on the post-transcriptional processing and mRNA maturation of the indicated glycolysis-related nuclear genes in A549 cells. RT reactions omitting reverse transcriptase were used as negative controls. (C) Native RIP followed by RT-qPCR detecting the binding of hnRNPA2B1 to HK2 or PFKFB3 pre-mRNA in control and lncCyt b-depleted A549 cells. The retrieved RNAs are presented as a percentage of the amount input. Data are shown as means ±SD of n = 3 independent experiments. ***P < 0.001 by Student’s *t* test.

We further defined the potential molecular mechanism through which lncCyt b regulates nuclear genes via the associated hnRNPA2B1. Knockdown of lncCyt b was found to decrease both the RNA and protein levels of *HK2* (Hexokinase 2) and *PFKFB3* (6-phosphofructo-2-kinase), two nuclear genes functionally implicated in glycolysis; also, hnRNPA2B1 depletion exhibited an equivalent effect on expression of the two nuclear genes (S11 Fig). A HITS-CLIP-seq data (GSM1716538) identified hnRNPA2B1 binding on the HK2 and PFKFB3 pre-mRNAs (Fig 6A). Thus, we wondered if lncCyt b can cooperate with hnRNPA2B1 to regulate HK2 and PFKFB3 at the post-transcriptional level. To test this notion, we designed primer sets for pre-mRNA and mature mRNA detection, respectively (Fig 6A). Our results showed that both hnRNPA2B1 and lncCyt b knockdown resulted in an increased level of pre-mRNAs while a decreased level of mature mRNAs (Fig 6B). Moreover, following the ASO-mediated lncCyt b knockdown, native RIP assay demonstrated a reduced hnRNPA2B1 binding on the HK2 and PFKFB3 pre-mRNAs in A549 cells (Fig 6C). Therefore, via the associated splicing factor hnRNPA2B1, the nuclear-translocated lncCyt b may regulate nuclear genes, including the glycolysis-related genes, by influencing their post-transcriptional processing and the following mRNA maturation.

## Discussion

Since an intense communication between nucleus and mitochondria is necessary for the homeostasis of whole cells, it is important to identify biological molecules that contribute to the inter-compartmental crosstalk. LncRNAs can be arbitrarily classified into nulncRNAs and mtlncRNAs according to their origin. In the RNA biology field, lncRNAs, especially nulncRNAs, have been extensively studied because of their implication in numerous biological events, and specific nulncRNAs have been involved in the regulation of mitochondrial function [28–32]. However, whether and how mtlncRNAs operate in the regulation of nuclear events remains largely elusive. In current study, several mtlncRNAs were identified to participate in the mitochondria-to-nucleus retrograde regulation. Mediated by the associated HuR and PNPT1, the mtlncRNAs MDL1AS, lncND5 and lncCyt b undergo a mitochondria-to-nucleus translocation. Moreover, as an example of its functional consequence, the nuclear-translocated lncCyt b was shown to cooperate with the splicing factor hnRNPA2B1 to influence several aspects of cell metabolism including glycolysis, possibly depending on their regulatory effect on related nuclear genes at the post-transcriptional level.

Nuclear genes influence mitochondrial activities via a mechanism of anterograde regulation. Conversely, mitochondrial signaling modulates nuclear events via a mechanism termed retrograde regulation. By acting as anterograde messengers, a number of nulncRNAs, such as CARL, Cerox1 and Tug1, exert direct or indirect regulatory effect on mitochondria-related events including apoptosis, energy metabolism and oxidative stress [33–35]. As for mtlncRNAs that act in the retrograde regulation, although relevant information is limited [36], our related study showed that the mtlncRNA MDL1 cooperates with Tid1 to inhibit the nuclear translocation of p53, a canonical transcription factor, thereby regulating a network of nuclear genes implicated in the cell cycle controlling [15].

It has been clear that the function of lncRNAs correlates tightly with their subcellular distribution. Somewhat consistent with this notion, the nuclear-encoded housekeeping tRNAs and 5S rRNA have long been realized to reside in mitochondria for the regulation of mitochondrial function [14], and certain nulncRNAs such as SAMMSON, SRA and GAS5 were proposed to play roles in specific cellular contexts after their import into mitochondria [28–31]. Moreover, the nulncRNA hTERC undergoes a bidirectional transportation between the nucleus and mitochondria, and then is processed to a shorter transcript that reflects the changes of mitochondrial function [37]. However, whether mtlncRNAs operate in the retrograde regulation on the basis of their nuclear translocation remains an issue to be addressed. To fill the knowledge gap, we analyzed the subcellular localization of a panel of mtlncRNAs. We demonstrated that the mtlncRNAs MDL1AS, lncND5 and lncCyt b exhibit a nuclear distribution, hinting that they undergo a mitochondria-to-nucleus translocation. Moreover, the nuclear distribution of MDL1AS, lncND5 and lncCyt b was detected in all cell types we tested, suggesting that their mitochondria-to-nucleus translocation should be a general event that can occur in different cellular contexts.

RNA binding proteins (RBPs) are well-known to bind and then determine the fate and function of their RNA targets [38]. Here, we identified both HuR and PNPT1 as protein partners that simultaneously associate with MDL1AS, lncND5 and lncCyt b. As a multifunctional RBP, HuR is widely implicated in the regulation of stability and translation of its target mRNAs; moreover, it was found to bind different RNA species, including protein-coding and noncoding transcripts, in the nucleus and then facilitate their nuclear export [20, 39]. PNPT1 exhibits a dual mitochondrial and cytoplasmic distribution [22, 23]. As for the mitochondria-targeted PNPT1, in addition to its involvement in RNA processing and polyadenylation in the mitochondrial matrix [22, 26], PNPT1 localized in the MIS was reported to facilitate import of the cytoplasmic RNAs into mitochondria [24]. Intriguingly, our current results showed that HuR and PNPT1 operate in a contrary direction to promote the nuclear translocation of the mtlncRNAs MDL1AS, lncND5 and lncCyt b. Furthermore, the overexpressed HuR- and PNPT1-binding segments, which can act as competitors to attenuate the reciprocal interaction between HuR/PNPT1 and the mtlncRNAs, were found to decrease the nuclear distribution of the mtlncRNAs. Together, our findings suggest that PNPT1 and HuR may contribute to the mitochondria-to-nucleus translocation of specific mtlncRNAs by facilitating their mitochondrial export and nuclear import, respectively.

We also investigated functional consequence of the mtlncRNAs after their nuclear translocation. Knockdown of the nuclear-translocated mtlncRNAs was revealed to influence a network of nuclear genes. In certain cases, rather than regulating their RNA targets, RBPs are inversely modulated by the bound RNA molecules [40]. In accordance with this notion, many lncRNAs are demonstrated to participate in the regulation of gene expression in ways of modulating activity of their protein partners, including factors implicated in epigenetic modification, RNA transcription, pre-mRNA processing and protein translation, among others [9]. Here, the splicing factor hnRNPA2B1 was identified as a protein partner of the nuclear-translocated lncCyt b. Moreover, lncCyt b was found to cooperate with hnRNPA2B1 to influence several aspects of cell metabolism including glycolysis, possibly depending on their synergistic effect on the post-transcriptional processing and mRNA maturation of related nuclear genes. Overall, our findings advance our knowledge in mitochondrial biology and provide new insights into the role of mtlncRNAs in mitochondria-nucleus communication.

## Materials and Methods

### Plasmid construction

For RNA interference, forward and reverse oligonucleotides were synthesized, annealed and cloned into the *Age* I/*Eco*R I sites of the pLKO.1 vector (Addgene). The segments of lncCyt b cDNA were synthesized from A549 cells by RT-PCR and cloned into the restriction sites *Eco*R I and *Xho* I of plasmid pLVX-Puro (BD Clontech). The oligonucleotides and primers are listed in S2 Table.

### Cell culture

A549, HEK293T, AGS, HCT116, HeLa, MDA-MB-231, BJ and HUVEC cells were obtained from the American Type Culture Collection (ATCC) and cultured in Dulbecco’s minimum essential medium (DMEM), RPMI 1640, or endothelial cell medium (ECM) supplemented with 10% fetal bovine serum (FBS) in a 5% CO2 incubator at 37°C. Plasmid transfection was performed using Lipofectamine 2000 (Invitrogen). For lentivirus infection, shRNA-encoding pLKO.1, or pLVX-Puro encoding lncCyt b segment, was co-transfected with psPAX2 and pMD2.G plasmids (Addgene) into HEK293 cells; the infectious lentivirus was harvested 2 days after transfection, filtered through 0.45-μm polyvinylidene difluoride (PVDF) filters, and transduced into specific cells. After plasmid transfection or lentivirus infection, the resulting cell population, but not the isolated single clones, was used for subsequent assays to avoid clone-specific effects.

### Subcellular fractionation

A total of 5 × 10^6^ cells were harvested by cell scraper and washed twice by cold PBS, and then were incubated in hypotonic buffer [50 mM HEPES (pH 7.4), 10 mM KCl, 350 mM sucrose, 1 mM EDTA, 1 mM DTT, 0.1% Triton X-100, 1 × Protease Inhibition Cocktail (PIC; Roche) and 100 U/ml RNase Inhibitor (Thermo Fisher Scientific)] on ice with frequent vortexing for 10 min. After 5 min of centrifugation at 2,000 *g*, the supernatant was collected as the cytoplasmic fraction, and after additional washing, the remainder was considered as nuclear pellets, which could be resuspended in lysis buffer [10 mM HEPES (pH 7.0), 100 mM KCl, 5 mM MgCl_2_, 0.5% NP-40, 10 μM DTT, 1 × PIC and 100 U/ml RNase Inhibitor] to prepare the nuclear lysate. For mitochondria isolation, cytoplasmic fraction was centrifugated at 12,000 *g* in 4°C for 15 min, the pellet was collected as mitochondrial fraction, the rest supernatant was collected as the cytoplasmic fraction without mitochondria.

### Gene expression analysis

The total RNA was isolated from cells by using RNAiso Plus reagent (Takara), treated with DNase I (Thermo Fisher Scientific), and then reverse-transcribed into cDNA using random hexamers or gene-specific primers with RT Easy I (Foregene). Gene expression levels were measured by qPCR using Real-Time PCR Easy (Foregene). The primers used in qPCR are shown in S2 Table. The high-throughput RNA-seq was conducted by Novogene Technology (Beijing, China). Genes with an adjusted *P*-value < 0.05 identified by DESeq2 were assigned as differentially expressed.

### RNA pull-down

To synthesize biotin-labeled MDL1AS, lncND5 and lncCyt b, PCR products were prepared using forward primers harboring the T7 RNA polymerase promoter. After purification of the PCR products, biotinylated transcripts were synthesized using the MaxiScript T7 Kit (Ambion). Biotinylated RNA (1 μg) was folded in RNA structure buffer [0.1 M KCl, 10 mM MgCl_2_, and 10 mM Tris-HCl (pH 7.0)] and then incubated with cell lysates at 25°C for 3 hrs with rotation. After incubation, RNA/protein complexes were isolated with streptavidin-coupled Dynabeads (Invitrogen). The retrieved RNA-associated proteins were fractionated by SDS-PAGE and visualized by silver staining. Protein bands present only in a specific RNA samples were excised and identified by mass spectrometry. The retrieved protein samples were confirmed by immunoblotting.

### Immunoblotting

A total of 1 × 10^5^ cells were washed twice in cold PBS and pelleted. The pellet was resuspended in lysis buffer [10 mM Tris-HCl (pH 7.4), 150 mM NaCl, 0.5% NP-40, 1 mM EDTA, 10 μM DTT, and 1 mM PMSF], incubated on ice with frequent vortexing for 15 min, and then the lysate was obtained by centrifugation at 12,000 *g* for 10 min. Protein concentrations of the extracts were measured by the bicinchoninic acid assay (Pierce). Four hundred micrograms of the protein was used for fractionated by SDS-PAGE, transferred onto PVDF membranes, and then blotted.

### Native RIP assay

A total of 1 × 10^7^ cells were washed twice in cold PBS and pelleted. The pellet was resuspended in lysis buffer [10 mM Tris-HCl (pH 7.4), 150 mM NaCl, 0.5% NP-40, 1 mM EDTA, 10 μM DTT, 50 U/ml RNase OUT (Invitrogen) and 1 × PIC], incubated on ice with frequent vortexing for 15 min, and then the lysate was obtained by centrifugation at 12,000 *g* for 10 min. After an incubation of the cell lysate with specific antibody or control IgG overnight at 4°C, samples were incubated with 50 μl of protein G magnetic beads for 1 hr at 4°C and then washed three times in lysis buffer. The beads were resuspended and treated with proteinase K at 45°C for 45 min. Coprecipitated RNAs were extracted using TRIzol reagent (Invitrogen), ethanol-precipitated with GlycoBlue (Invitrogen) as a carrier, and then detected by RT-qPCR. The data of retrieved RNAs are presented as a percentage of the amount input.

For RIP-based mapping assay, cell lysates were mixed with RNase T1 (1 U/ml, Thermo Fisher Scientific), after which standard native RIP assays were performed. Following extraction of the coprecipitated RNA, RNA segments bound by specific proteins, and hence protected from RNase T1 digestion and immunoprecipitated, were identified by RT-qPCR analysis using primer sets that scanned the entire mtlncRNA sequence at ~170 nt-long, overlapping intervals. The primers used for RT-qPCR following native RIP and RIP-based mapping assays are shown in S2 Table.

### RNase R treatment for circRNA detection

For RNase R treatment, 5 μg total RNA was treated with 8 U RNase R (Beyotime) in 50 μl total volume for 30 min, and the resultant RNA samples were subjected to cDNA synthesis using random hexamer primers and the following PCR amplification with gene-specific convergent or divergent primers. The convergent or divergent primers are shown in S2 Table.

### Quantification of lncRNA copy number per cell

DNA fragments corresponding to human MDL1AS, lncND5, lncCyt b, MALAT1 and XIST were amplified through RT-PCR reaction with specific primers. The purified DNA fragments were diluted by multiple grads to plot standard curves through real-time PCR. Total RNAs from 1.6 × 10^6^ A549 cells were prepared for cDNA synthesis using random hexamer primers, and cDNAs were subjected to qPCR with specific primers. The qPCR primers used for quantification of lncRNA copy number are shown in S2 Table.

### Antibodies

The antibodies used for RNA pull-down, native RIP and immunoblotting were anti-ACTB (66009-1-Ig, Proteintech), anti-HuR (11910-1-AP, Proteintech), anti-hnRNPA2B1 (14813-1-AP, Proteintech), anti-PNPT1 (14487-1-AP, Proteintech), anti-Hexokinase 1 (19662-1-AP, Proteintech), anti-Hexokinase 2 (22029-1-AP, Proteintech), anti-PFKFB3 (13763-1-AP, Proteintech). anti-IGF2BP1(22803-1-AP, Proteintech)

### ASOs

The antisense oligonucleotides (ASOs) were purchased from RIBOBIO company (Guang Zhou, China). The ASO-targeting sequences within the mtlncRNAs were shown in S3 Table.

### RNA FISH

RNA FISH was performed using QuantiGene ViewRNA ISH Cell Assay Kit (Thermo Fisher Scientific) according to the manufacturer’s instructions. Briefly, cells cultured on coverslips were fixed, permeabilized, and digested by protease to allow target accessibility, and then specific probe sets (designed and supplied by Thermo Fisher Scientific) was added to the cells and hybridization was performed at 40°C for 3 hrs. After a series of signal amplification with reagents supplied in the kit, cells were counterstained with DAPI and then detected using an inverted confocal microscope (Leica).

### Glycolysis stress test

The extracellular acidification rate (ECAR) was analyzed using Seahorse XFp Extracellular Flux Analyzer (Agilent Seahorse Bioscience). Cells were seeded at 5 × 10^4^ cells per well in an XF96 cell culture microplate and maintained for 24 hrs. Prior to ECAR analysis, the culture media was replaced with Seahorse XF DMEM medium (pH 7.4, without glucose and glutamine) and cells were allowed to equilibrate in a CO_2_-free incubator. ECAR was measured after supplementation of 100 mM glucose, 15 μM oligomycin and 500 mM 2-deoxyglucose (2-DG). ECAR values were normalized to cell numbers.

### Cell proliferation assay

A total of 2,000 cells were seeded into 96-well plates. At each time point, the culture medium was replaced with CCK-8 solution (Beyotime), and then the cells were incubated at 37°C for 1 hr. The absorbance values were read at 450 nm using a Thermo Fisher Scientific Varioskan Flash multimode reader, and the cell proliferation curves were plotted using the absorbance at each time point.

### Transwell migration and invasion assay

For the migration assays, 24-well, 8-μm-diameter micropore membranes without Matrigel transwell plates (Mllipore) was used. Invasion assays were conducted using 8-μm-pore, 24-well transwell plates with Matrigel (Corning). A total of 5 × 10^4^ resuspended cells were seeded into upper compartment of migration or invasion chambers, with the bottom chamber was filled with 600 μl of culture medium with 20% FBS as an attractant. After incubation in 5% CO_2_ at 37°C for 48 hrs, cells that had migrated or invaded through the membrane were fixed with 4% paraformaldehyde, stained with a crystal violet, and imaged under a microscope to determine cell numbers.

### Co-IP

A total of 1 × 10^7^ cells were washed three times in cold PBS and pelleted. The pellet was resuspended in IP lysis buffer [10mM tris-HCl (pH7.4), 150 mM NaCl, 0.5% NP-40, 1mM EDTA, 10% Glycerol and 1mM PMSF], incubated on ice for 15 min with frequent vortexing, and then the lysate was obtained by centrifugation at 12,000 *g* for 10 min. Protein concentration of the extracts was measured by the bicinchoninic acid assay (Pierce). Four hundred micrograms of the protein samples were incubated with a specific antibody or control IgG overnight at 4°C. Subsequently, the samples were incubated with 50 ul of protein G magnetic beads (Thermo Fisher Scientific) for 2 hrs at 4°C and then washed three times in IP lysis buffer. Last, protein complexes were eluted in SDS loading buffer [10% SDS, 50 mM tris-HCl (pH6.8), 10% glycerol, 1% β-mercaptoethanol and 0.1% bromophenol blue] and then detected by immunoblotting.

### ELISA assays for the RNA-protein interaction

HuR or PNPT1 proteins (500 ng/well) were coated in the 96-well ELISA microplates with 100 μl coating buffer [100 mM NaHCO_3_ (pH = 9.6)] at 4°C overnight. After twice washing with washing buffer [0.55 mM MgCl_2_, 0.2% Tween-20 in PBS], the plates were subjected to 1 hr of blocking at room temperature (RT) with 200 μl of blocking buffer [PBST containing 2% BSA]. Then, serial dilutions (200, 400, 600, 800, 1000 nM) of biotin-labeled oligonucleotides were added into the wells and incubated in binding buffer [0.55 mM MgCl_2_ in PBS] at RT for 30 min with 80 rpm shaking. After twice washing, streptavidinhorseradish peroxidase (HRP) and its substrates were sequentially added to the reactions. Color development was carried out using 3,3′,5,5′- Tetramethylbenzidine (TMB) substrate and measured using a microplate reader at 450 nm absorbance.

For competitive ELISA assays, after the common procedures described above, 800 nM biotin-labeled oligonucleotides (MDL1AS, lncND5 and lncCyt b) and serial dilutions (200, 400, 600, 800, 1000 nM) of unlabeled oligonucleotides were added into microplates and incubated at RT for 30 min with 80 rpm shaking. Then, HRP-conjugated streptavidin and TMB substrate were used to measure the absorbance at 450 nm on a microplate reader.

### Statistical analysis

Statistical analyses of experimental data were performed using the GraphPad Prism software. Student’s *t* test was performed as indicated to compare the differences between experimental groups relative to their paired controls. The data were presented as the means ± SD, and *P* < 0.05 or below was considered statistically significant.

## Supporting information

S1 Fig Several mtlncRNAs undergo a mitochondria-to-nucleus translocation. (A) The map of human mtDNA. (B) RT-qPCR assay following nuclear/cytoplasmic fractionation detecting the distribution of the indicated mitochondrial RNAs in AGS, BJ, and HUVEC cells. U2 snRNA, a canonical nuclear-retained transcript, and the mitochondrial 12S and 16S rRNAs, were assessed as controls to confirm the findings of our nuclear/cytoplasmic fractionation. Data are shown as means ± SD of n = 3 independent experiments. (C) RT-PCR assays for distribution of the indicated transcripts in the nuclear and cytoplasmic fractions of a panel of cell lines. Total extracts and RT reactions omitting reverse transcriptase were used as controls.
(TIFF)

S2 Fig Molecular features of the nuclear-translocated mtlncRNAs. (A) RNA FISH detecting distribution of the indicated mtlncRNAs in AGS, BJ, and HUVEC cells. Scale bars, 10 μm. (B) RT-PCR with divergent and convergent primers for linear and circular RNA detection in A549 cells. ACTB mRNA and mecciND1 were included as a linear and circRNA control, respectively. RT reaction omitting reverse transcriptase was included as a negative control. (C) Absolute quantitation assay for MDL1AS, lncND5 and lncCyt b transcripts within the nucleus and cytoplasm of A549 cells. MALAT1 and XIST, two lncRNAs demonstrated to be expressed at a high or low level, were included as controls.
(TIFF)

S3 Fig HuR facilitates nuclear translocation of specific mtlncRNAs. (A) Silver staining of proteins pulled down with the indicated RNA baits in A549 cells. Red arrow denotes the protein bands excised and subjected to mass spectrometry analysis. (B) Immunoblot detecting the HuR levels in the proteins pulled down by the indicated RNA baits. (C) Immunoblot detecting the shRNA-mediated HuR knockdown in A549 cells. (D) Nuclear/cytoplasmic fractionation followed by RT-qPCR detecting the influence of HuR knockdown on nuclear distribution of the indicated RNA transcripts in A549 cells. Data are shown as means ± SD of n = 3 independent experiments. ***P < 0.001 by Student’s *t* test. (E) RT-qPCR detecting the influence of HuR knockdown on the stability of MDL1AS, lncND5, and lncCyt b, with the Fos mRNA being tested as the control [21]. Data are shown as means ± SD of n = 3 independent experiments. *P < 0.05, ***P < 0.001 by Student’s *t* test; ns, not significant.
(TIFF)

S4 Fig HuR facilitates nuclear translocation of mtlncRNAs via its binding to specific mtlncRNA regions. (A) ELISA assays detecting the association of HuR protein with different regions of MDL1AS, lncND5 and lncCyt b. Data are shown as means ± SD of n = 3 independent experiments. (B) RT-qPCR detecting overexpression of the indicated lncCyt b segments in A549 cells. Data are shown as means ± SD of n = 3 independent experiments. ***P < 0.001 by Student’s *t* test. (C) Nuclear/cytoplasmic fractionation followed by RT-qPCR detecting the distribution of U2 snRNA and 12S rRNAs to confirm the findings of our nuclear/cytoplasmic fractionation in Fig 3B. Data are shown as means ± SD of n = 3 independent experiments. (D) Competitive ELISA assays detecting effect of the lncCyt b segment F1 or F7 on HuR association with MDL1AS, lncND5 and lncCyt b. Data are shown as means ± SD of n = 3 independent experiments.
(TIFF)

S5 Fig PNPT1 regulates the subcellular distribution of mtlncRNAs. (A) Immunoblot detecting the PNPT1 level in the proteins pulled down by the indicated RNA baits. (B) Immunoblot detecting the shRNA-mediated PNPT1 knockdown in A549 cells. (C) Nuclear/cytoplasmic fractionation followed by RT-qPCR detecting the influence of PNPT1 knockdown on nuclear distribution of the indicated RNA transcripts in A549 cells. Data are shown as means ± SD of n = 3 independent experiments. **P < 0.01, ***P < 0.001 by Student’s *t* test. (D) ELISA assays detecting the association of PNPT1 protein with different regions of MDL1AS, lncND5 and lncCyt b. Data are shown as means ± SD of n = 3 independent experiments. (E) Competitive ELISA assays detecting effect of the lncCyt b segment F1 or F3 on PNPT1 association with MDL1AS, lncND5 and lncCyt b. Data are shown as means ± SD of n = 3 independent experiments.
(TIFF)

S6 Fig PNPT1, rather than mtPAP, contributes to the nuclear translocation of mtlncRNAs. (A) Co-IP assay with anti-HuR antibody detecting the HuR/PNPT1 interaction in whole-cell lysates of A549 cells. IGF2BP1 was included as a control of the HuR-binding partner. (B) Immunoblot detecting the shRNA-mediated mtPAP knockdown in A549 cells. (C) Nuclear/cytoplasmic fractionation followed by RT-qPCR detecting the distribution of U2 snRNA and 12S rRNAs to confirm the findings of our nuclear/cytoplasmic fractionation in (D). Data are shown as means ± SD of n = 3 independent experiments. (D) Nuclear/cytoplasmic fractionation followed by RT-qPCR detecting the influence of mtPAP knockdown on the nuclear distribution of MDL1AS, lncND5 and lncCyt b in A549 cells. Data are shown as means ± SD of n = 3 independent experiments. N.S, not significant.
(TIFF)

S7 Fig RT-qPCR validating the downregulation of nuclear-localized MDL1AS, lncND5 and lncCyt b by two independent ASOs in A549 cells. Data are shown as means ± SD of n = 3 independent experiments. ***P < 0.001 by Student’s *t* test.
(TIFF)

S8 Fig The nuclear-translocated mtlncRNAs modulate a network of nuclear genes. (A) Heatmaps showing the changes in nuclear gene expression compared with the negative control after knockdown of MDL1AS, lncND5 and lncCyt b with two independent ASOs. (B) GO enrichment analysis of the nuclear genes regulated by MDL1AS, lncND5 and lncCyt b. (C) RT-qPCR validating nuclear genes regulated by MDL1AS, lncND5 and lncCyt b. Specialized biological functions of the nuclear genes are annotated. Data are shown as means ± SD of n = 3 independent experiments. **P < 0.01, ***P < 0.001 by Student’s *t* test.
(TIF)

S9 Fig lncCyt b enhances the stability of hnRNPA2B1 protein. (A) Immunoblot detecting influence of lncCyt b knockdown on the hnRNPA2B1 protein level in A549 cells. (B) RT-qPCR detecting influence of lncCyt b knockdown on the hnRNPA2B1 mRNA level in A549 cells. (C) Immunoblot detecting the hnRNPA2B1 protein level in control and lncCyt b-depleted A549 cells after the treatment with CHX, MG132 or CQ at the indicated time points.
(TIFF)

S10 Fig lncCyt b and hnRNPA2B1 exhibit physiological significance. (A) CCK-8 assays showing the deceased proliferation of HCT116, MDA-MB-231 and HeLa cells by lncCyt b or hnRNPA2B1 knockdown. Data are shown as means ± SD of n = 3 independent experiments. ***P < 0.001 by Student’s *t* test. (B) Transwell assays showing the weakened migration and invasion of HCT116, MDA-MB-231 and HeLa cells by lncCyt b or hnRNPA2B1 knockdown. Scale bars, 100 μm.
(TIFF)

S11 Fig lncCyt b and hnRNPA2B1 modulate the glycolysis-related nuclear genes. (A) RT-qPCR detecting influence of lncCyt b or hnRNPA2B1 knockdown on the RNA level of the indicated glycolysis-related genes. Data are shown as means ± SD of n = 3 independent experiments. **P < 0.01, ***P < 0.001 by Student’s *t* test. (B) Immunoblot detecting influence of lncCyt b or hnRNPA2B1 knockdown on the protein level of the indicated glycolysis-related genes.
(TIFF)

S1 Table Protein partners of the mtlncRNAs MDL1AS, lncND5 and lncCyt b identified by RNA pull-down followed by mass spectrometry from A549 cells. (XLSX)

S2 Table Oligonucleotides or primers used in this study. (XLSX)

S3 Table The mtlncRNA-targeting ASOs used in this study. (XLSX)

S4 Table Raw data used for the figures in main text. (XLSX)

S5 Table Raw data used for the figures in supplementary information. (XLSX)

## References

[1] Gray MW, Burger G, Lang BF. Mitochondrial evolution. Science. 1999;283(5407):1476–81. Epub 1999/03/05. doi: 10.1126/science.283.5407.1476 . 10066161

[2] Lane N, Martin W. The energetics of genome complexity. Nature. 2010;467(7318):929–34. Epub 2010/10/22. doi: 10.1038/nature09486 . 20962839

[3] Schmidt O, Pfanner N, Meisinger C. Mitochondrial protein import: from proteomics to functional mechanisms. Nat Rev Mol Cell Biol. 2010;11(9):655–67. Epub 2010/08/24. doi: 10.1038/nrm2959 . 20729931

[4] Richter-Dennerlein R, Dennerlein S, Rehling P. Integrating mitochondrial translation into the cellular context. Nat Rev Mol Cell Biol. 2015;16(10):586–92. Epub 2015/11/05. doi: 10.1038/nrm4051 . 26535422

[5] Bock FJ, Tait SWG. Mitochondria as multifaceted regulators of cell death. Nat Rev Mol Cell Biol. 2020;21(2):85–100. Epub 2019/10/23. doi: 10.1038/s41580-019-0173-8 . 31636403

[6] Spinelli JB, Haigis MC. The multifaceted contributions of mitochondria to cellular metabolism. Nat Cell Biol. 2018;20(7):745–54. Epub 2018/06/29. doi: 10.1038/s41556-018-0124-1 ; PubMed Central PMCID: PMC6541229. 29950572 PMC6541229

[7] Quiros PM, Mottis A, Auwerx J. Mitonuclear communication in homeostasis and stress. Nat Rev Mol Cell Biol. 2016;17(4):213–26. Epub 2016/03/10. doi: 10.1038/nrm.2016.23 . 26956194

[8] Mottis A, Herzig S, Auwerx J. Mitocellular communication: Shaping health and disease. Science. 2019;366(6467):827–32. Epub 2019/11/16. doi: 10.1126/science.aax3768 . 31727828

[9] Statello L, Guo CJ, Chen LL, Huarte M. Gene regulation by long non-coding RNAs and its biological functions. Nat Rev Mol Cell Biol. 2021;22(2):96–118. Epub 2020/12/24. doi: 10.1038/s41580-020-00315-9 ; PubMed Central PMCID: PMC7754182. 33353982 PMC7754182

[10] Vendramin R, Marine JC, Leucci E. Non-coding RNAs: the dark side of nuclear-mitochondrial communication. Embo J. 2017;36(9):1123–33. Epub 2017/03/21. doi: 10.15252/embj.201695546 ; PubMed Central PMCID: PMC5412819. 28314780 PMC5412819

[11] Gusic M, Prokisch H. ncRNAs: New Players in Mitochondrial Health and Disease? Front Genet. 2020;11:95. Epub 2020/03/18. doi: 10.3389/fgene.2020.00095 ; PubMed Central PMCID: PMC7059738. 32180794 PMC7059738

[12] Mercer TR, Neph S, Dinger ME, Crawford J, Smith MA, Shearwood AM, et al. The human mitochondrial transcriptome. Cell. 2011;146(4):645–58. Epub 2011/08/23. doi: 10.1016/j.cell.2011.06.051 ; PubMed Central PMCID: PMC3160626. 21854988 PMC3160626

[13] Gao S, Tian X, Chang H, Sun Y, Wu Z, Cheng Z, et al. Two novel lncRNAs discovered in human mitochondrial DNA using PacBio full-length transcriptome data. Mitochondrion. 2018;38:41–7. Epub 2017/08/15. doi: 10.1016/j.mito.2017.08.002 . 28802668

[14] Cannon MV, Irwin MH, Pinkert CA. Mitochondrially-imported RNA in drug discovery. Drug Dev Res. 2015;76(2):61–71. Epub 2015/04/08. doi: 10.1002/ddr.21241 . 25847616

[15] Li J, Bai R, Yang W, Miao H, Li Y, Dai H, et al. The mitochondrial-derived lncRNA MDL1 mediates a mitochondria-to-nucleus retrograde regulation by inhibiting the nuclear translocation of p53. MedComm–Oncology. 2022;1. doi: 10.1002/mog2.15

[16] Liu X, Wang XL, Li JX, Hu SS, Deng YQ, Yin H, et al. Identification of mecciRNAs and their roles in the mitochondrial entry of proteins. Sci China Life Sci. 2020;63(10):1429–49. doi: 10.1007/s11427-020-1631-9 WOS:000518376000001. 32048164

[17] Brennan CM, Steitz JA. HuR and mRNA stability. Cell Mol Life Sci. 2001;58(2):266–77. Epub 2001/04/06. doi: 10.1007/PL00000854 . 11289308 PMC11146503

[18] Gallouzi IE, Steitz JA. Delineation of mRNA export pathways by the use of cell-permeable peptides. Science. 2001;294(5548):1895–901. Epub 2001/12/01. doi: 10.1126/science.1064693 . 11729309

[19] Prechtel AT, Chemnitz J, Schirmer S, Ehlers C, Langbein-Detsch I, Stulke J, et al. Expression of CD83 is regulated by HuR via a novel cis-active coding region RNA element. J Biol Chem. 2006;281(16):10912–25. Epub 2006/02/18. doi: 10.1074/jbc.M510306200 . 16484227

[20] Noh JH, Kim KM, Abdelmohsen K, Yoon JH, Panda AC, Munk R, et al. HuR and GRSF1 modulate the nuclear export and mitochondrial localization of the lncRNA RMRP. Genes Dev. 2016;30(10):1224–39. Epub 2016/05/21. doi: 10.1101/gad.276022.115 ; PubMed Central PMCID: PMC4888842. 27198227 PMC4888842

[21] Peng SS, Chen CY, Xu N, Shyu AB. RNA stabilization by the AU-rich element binding protein, HuR, an ELAV protein. Embo J. 1998;17(12):3461–70. doi: 10.1093/emboj/17.12.3461 WOS:000074363800023. 9628881 PMC1170682

[22] Chen HW, Rainey RN, Balatoni CE, Dawson DW, Troke JJ, Wasiak S, et al. Mammalian polynucleotide phosphorylase is an intermembrane space RNase that maintains mitochondrial homeostasis. Mol Cell Biol. 2006;26(22):8475–87. Epub 2006/09/13. doi: 10.1128/MCB.01002-06 ; PubMed Central PMCID: PMC1636764. 16966381 PMC1636764

[23] Sarkar D, Park ES, Emdad L, Randolph A, Valerie K, Fisher PB. Defining the domains of human polynucleotide phosphorylase (hPNPaseOLD-35) mediating cellular senescence. Mol Cell Biol. 2005;25(16):7333–43. Epub 2005/08/02. doi: 10.1128/MCB.25.16.7333-7343.2005 ; PubMed Central PMCID: PMC1190265. 16055741 PMC1190265

[24] Wang G, Chen HW, Oktay Y, Zhang J, Allen EL, Smith GM, et al. PNPASE regulates RNA import into mitochondria. Cell. 2010;142(3):456–67. Epub 2010/08/10. doi: 10.1016/j.cell.2010.06.035 ; PubMed Central PMCID: PMC2921675. 20691904 PMC2921675

[25] Fuke H, Ohno M. Role of poly (A) tail as an identity element for mRNA nuclear export. Nucleic Acids Res. 2008;36(3):1037–49. Epub 2007/12/22. doi: 10.1093/nar/gkm1120 ; PubMed Central PMCID: PMC2241894. 18096623 PMC2241894

[26] Slomovic S, Schuster G. Stable PNPase RNAi silencing: its effect on the processing and adenylation of human mitochondrial RNA. Rna. 2008;14(2):310–23. Epub 2007/12/18. doi: 10.1261/rna.697308 ; PubMed Central PMCID: PMC2212247. 18083837 PMC2212247

[27] Tomecki R, Dmochowska A, Gewartowski K, Dziembowski A, Stepien PP. Identification of a novel human nuclear-encoded mitochondrial poly(A) polymerase. Nucleic Acids Res. 2004;32(20):6001–14. Epub 2004/11/18. doi: 10.1093/nar/gkh923 ; PubMed Central PMCID: PMC534615. 15547249 PMC534615

[28] Colley SM, Leedman PJ. SRA and its binding partners: an expanding role for RNA-binding coregulators in nuclear receptor-mediated gene regulation. Crit Rev Biochem Mol Biol. 2009;44(1):25–33. Epub 2009/03/13. doi: 10.1080/10409230802661719 . 19280430

[29] Vendramin R, Verheyden Y, Ishikawa H, Goedert L, Nicolas E, Saraf K, et al. SAMMSON fosters cancer cell fitness by concertedly enhancing mitochondrial and cytosolic translation. Nat Struct Mol Biol. 2018;25(11):1035–46. Epub 2018/10/31. doi: 10.1038/s41594-018-0143-4 ; PubMed Central PMCID: PMC6223542. 30374086 PMC6223542

[30] Leucci E, Vendramin R, Spinazzi M, Laurette P, Fiers M, Wouters J, et al. Melanoma addiction to the long non-coding RNA SAMMSON. Nature. 2016;531(7595):518–22. Epub 2016/03/25. doi: 10.1038/nature17161 . 27008969

[31] Sang L, Ju HQ, Yang Z, Ge Q, Zhang Z, Liu F, et al. Mitochondrial long non-coding RNA GAS5 tunes TCA metabolism in response to nutrient stress. Nat Metab. 2021;3(1):90–106. Epub 2021/01/06. doi: 10.1038/s42255-020-00325-z . 33398195

[32] Hermanns P, Bertuch AA, Bertin TK, Dawson B, Schmitt ME, Shaw C, et al. Consequences of mutations in the non-coding RMRP RNA in cartilage-hair hypoplasia. Hum Mol Genet. 2005;14(23):3723–40. Epub 2005/10/29. doi: 10.1093/hmg/ddi403 . 16254002

[33] Wang K, Long B, Zhou LY, Liu F, Zhou QY, Liu CY, et al. CARL lncRNA inhibits anoxia-induced mitochondrial fission and apoptosis in cardiomyocytes by impairing miR-539-dependent PHB2 downregulation. Nat Commun. 2014;5:3596. Epub 2014/04/09. doi: 10.1038/ncomms4596 . 24710105

[34] Sirey TM, Roberts K, Haerty W, Bedoya-Reina O, Rogatti-Granados S, Tan JY, et al. The long non-coding RNA Cerox1 is a post transcriptional regulator of mitochondrial complex I catalytic activity. Elife. 2019;8. Epub 2019/05/03. doi: 10.7554/eLife.45051 ; PubMed Central PMCID: PMC6542586. 31045494 PMC6542586

[35] Long J, Badal SS, Ye Z, Wang Y, Ayanga BA, Galvan DL, et al. Long noncoding RNA Tug1 regulates mitochondrial bioenergetics in diabetic nephropathy. J Clin Invest. 2016;126(11):4205–18. Epub 2016/11/02. doi: 10.1172/JCI87927 ; PubMed Central PMCID: PMC5096930. 27760051 PMC5096930

[36] Huang J, Wu S, Wang P, Wang G. Non-coding RNA Regulated Cross-Talk Between Mitochondria and Other Cellular Compartments. Front Cell Dev Biol. 2021;9:688523. Epub 2021/08/21. doi: 10.3389/fcell.2021.688523 ; PubMed Central PMCID: PMC8369480. 34414182 PMC8369480

[37] Cheng Y, Liu P, Zheng Q, Gao G, Yuan J, Wang P, et al. Mitochondrial Trafficking and Processing of Telomerase RNA TERC. Cell Rep. 2018;24(10):2589–95. Epub 2018/09/06. doi: 10.1016/j.celrep.2018.08.003 . 30184494

[38] Keene JD. RNA regulons: coordination of post-transcriptional events. Nat Rev Genet. 2007;8(7):533–43. Epub 2007/06/19. doi: 10.1038/nrg2111 . 17572691

[39] Wu M, Tong CWS, Yan W, To KKW, Cho WCS. The RNA Binding Protein HuR: A Promising Drug Target for Anticancer Therapy. Curr Cancer Drug Targets. 2019;19(5):382–99. Epub 2018/11/02. doi: 10.2174/1568009618666181031145953 . 30381077

[40] Li L, Miao H, Chang Y, Yao H, Zhao Y, Wu F, et al. Multidimensional crosstalk between RNA-binding proteins and noncoding RNAs in cancer biology. Semin Cancer Biol. 2021;75:84–96. Epub 2021/03/17. doi: 10.1016/j.semcancer.2021.03.007 . 33722631

