# Supplementary figures and images for: A mitochondria-to-nucleus regulation mediated by the nuclear-translocated mitochondrial lncRNAs
Source: PLoS Genet. 2025 Jan 27;21(1):e1011580. doi: 10.1371/journal.pgen.1011580 (PMC11801721; doi:10.1371/journal.pgen.1011580)

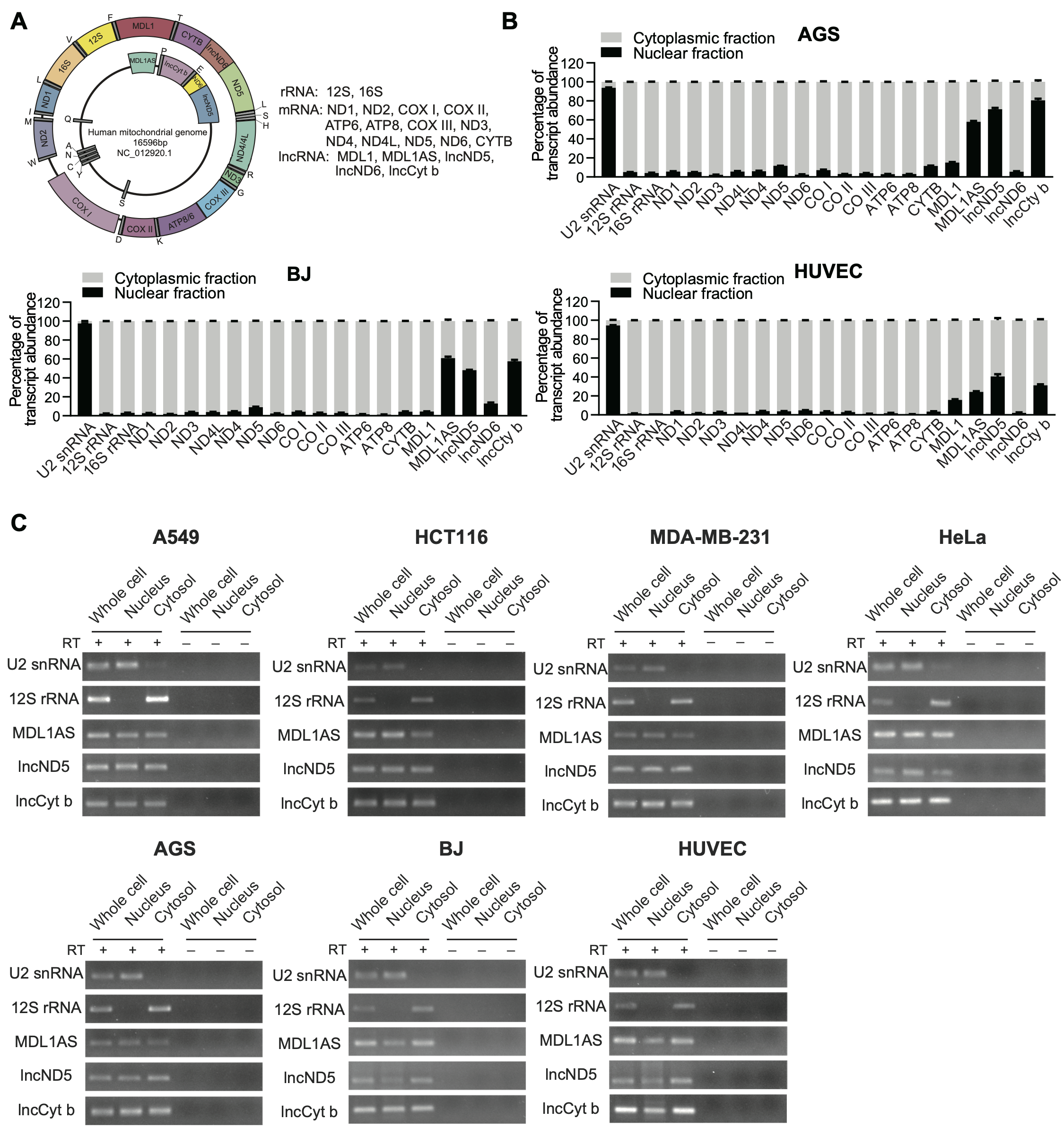

Supplement: S1 Fig — (A) The map of human mtDNA. (B) RT-qPCR assay following nuclear/cytoplasmic fractionation detecting the distribution of the indicated mitochondrial RNAs in AGS, BJ, and HUVEC cells. U2 snRNA, a canonical nuclear-retained transcript, and the mitochondrial 12S and 16S rRNAs, were assessed as controls to confirm the findings of our nuclear/cytoplasmic fractionation. Data are shown as means ± SD of n = 3 independent experiments. (C) RT-PCR assays for distribution of the indicated transcripts in the nuclear and cytoplasmic fractions of a panel of cell lines. Total extracts and RT reactions omitting reverse transcriptase were used as controls. (TIFF) [file pgen.1011580.s001.tiff]

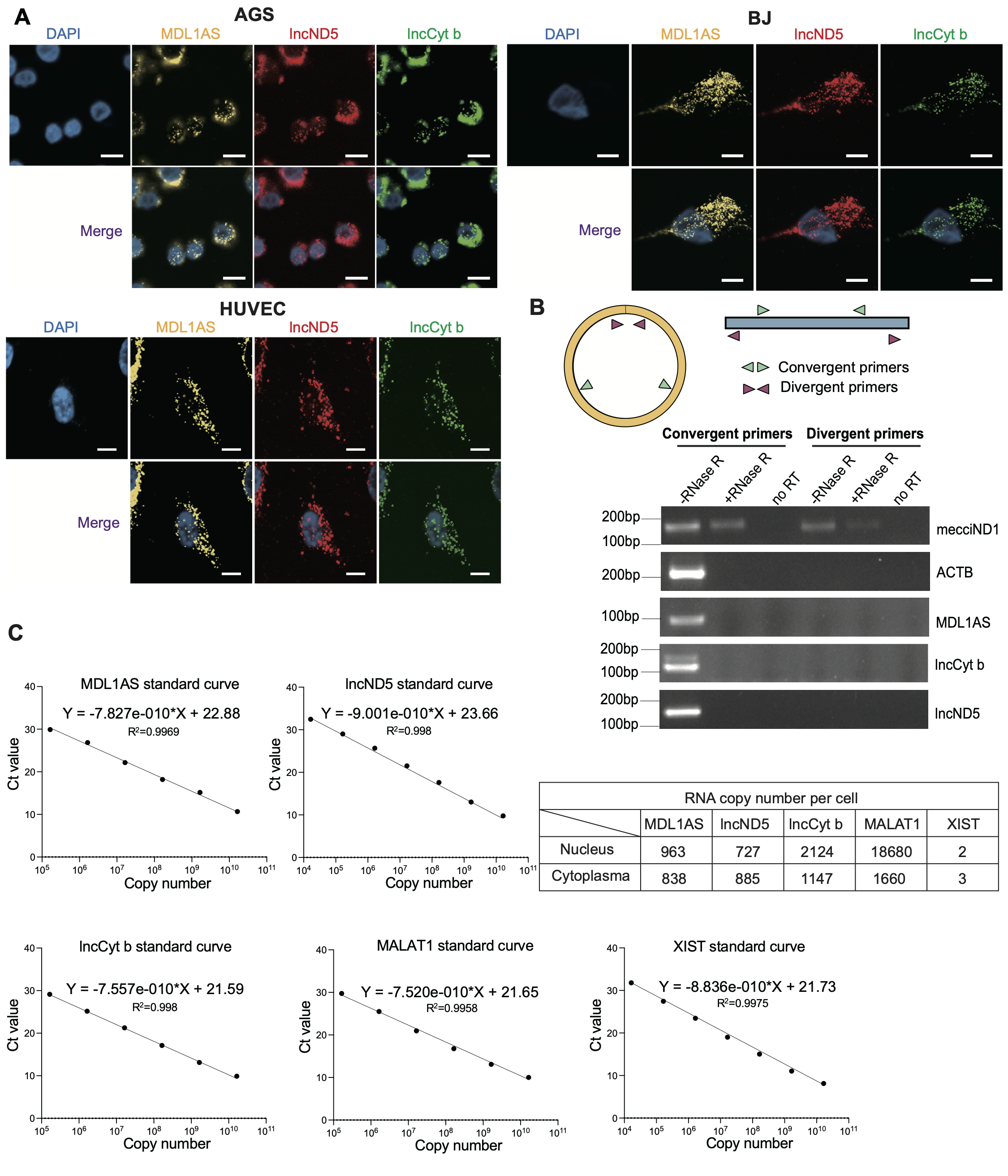

Supplement: S2 Fig — (A) RNA FISH detecting distribution of the indicated mtlncRNAs in AGS, BJ, and HUVEC cells. Scale bars, 10 μm. (B) RT-PCR with divergent and convergent primers for linear and circular RNA detection in A549 cells. ACTB mRNA and mecciND1 were included as a linear and circRNA control, respectively. RT reaction omitting reverse transcriptase was included as a negative control. (C) Absolute quantitation assay for MDL1AS, lncND5 and lncCyt b transcripts within the nucleus and cytoplasm of A549 cells. MALAT1 and XIST, two lncRNAs demonstrated to be expressed at a high or low level, were included as controls. (TIFF) [file pgen.1011580.s002.tiff]

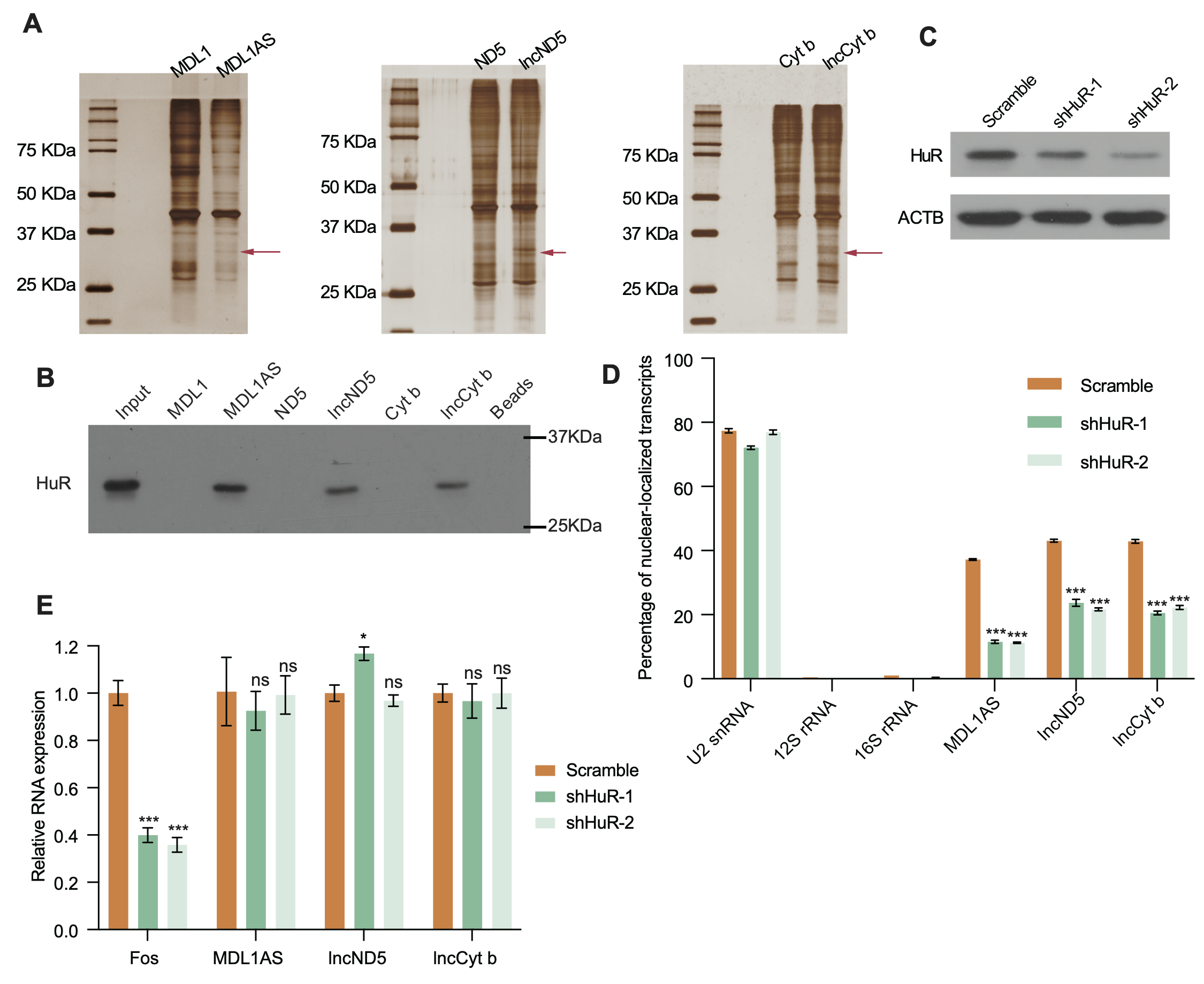

Supplement: S3 Fig — (A) Silver staining of proteins pulled down with the indicated RNA baits in A549 cells. Red arrow denotes the protein bands excised and subjected to mass spectrometry analysis. (B) Immunoblot detecting the HuR levels in the proteins pulled down by the indicated RNA baits. (C) Immunoblot detecting the shRNA-mediated HuR knockdown in A549 cells. (D) Nuclear/cytoplasmic fractionation followed by RT-qPCR detecting the influence of HuR knockdown on nuclear distribution of the indicated RNA transcripts in A549 cells. Data are shown as means ± SD of n = 3 independent experiments. ***P < 0.001 by Student’s t test. (E) RT-qPCR detecting the influence of HuR knockdown on the stability of MDL1AS, lncND5, and lncCyt b, with the Fos mRNA being tested as the control [21]. Data are shown as means ± SD of n = 3 independent experiments. *P < 0.05, ***P < 0.001 by Student’s t test; ns, not significant. (TIFF) [file pgen.1011580.s003.tiff]

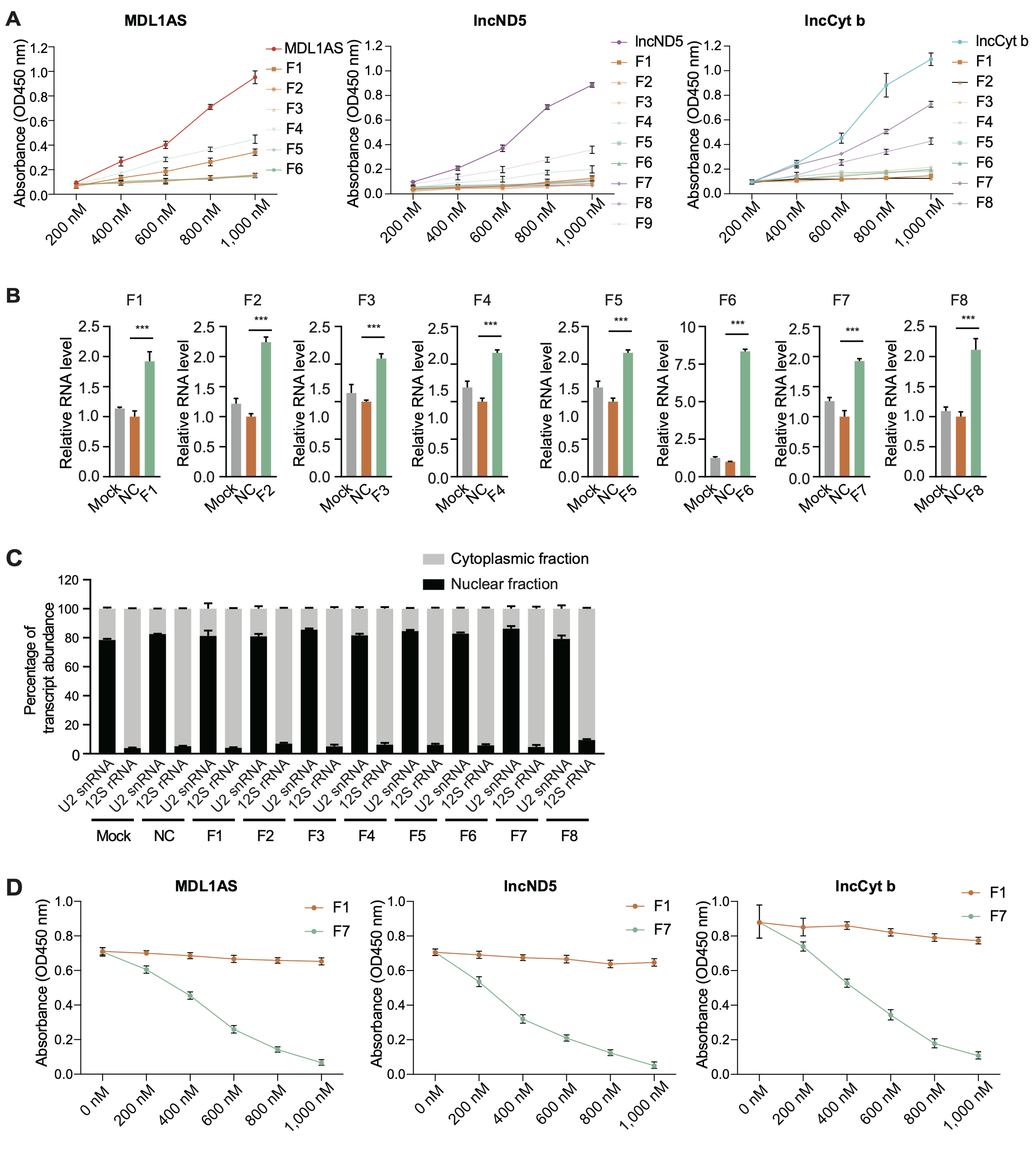

Supplement: S4 Fig — (A) ELISA assays detecting the association of HuR protein with different regions of MDL1AS, lncND5 and lncCyt b. Data are shown as means ± SD of n = 3 independent experiments. (B) RT-qPCR detecting overexpression of the indicated lncCyt b segments in A549 cells. Data are shown as means ± SD of n = 3 independent experiments. ***P < 0.001 by Student’s t test. (C) Nuclear/cytoplasmic fractionation followed by RT-qPCR detecting the distribution of U2 snRNA and 12S rRNAs to confirm the findings of our nuclear/cytoplasmic fractionation in Fig 3B. Data are shown as means ± SD of n = 3 independent experiments. (D) Competitive ELISA assays detecting effect of the lncCyt b segment F1 or F7 on HuR association with MDL1AS, lncND5 and lncCyt b. Data are shown as means ± SD of n = 3 independent experiments. (TIFF) [file pgen.1011580.s004.tiff]

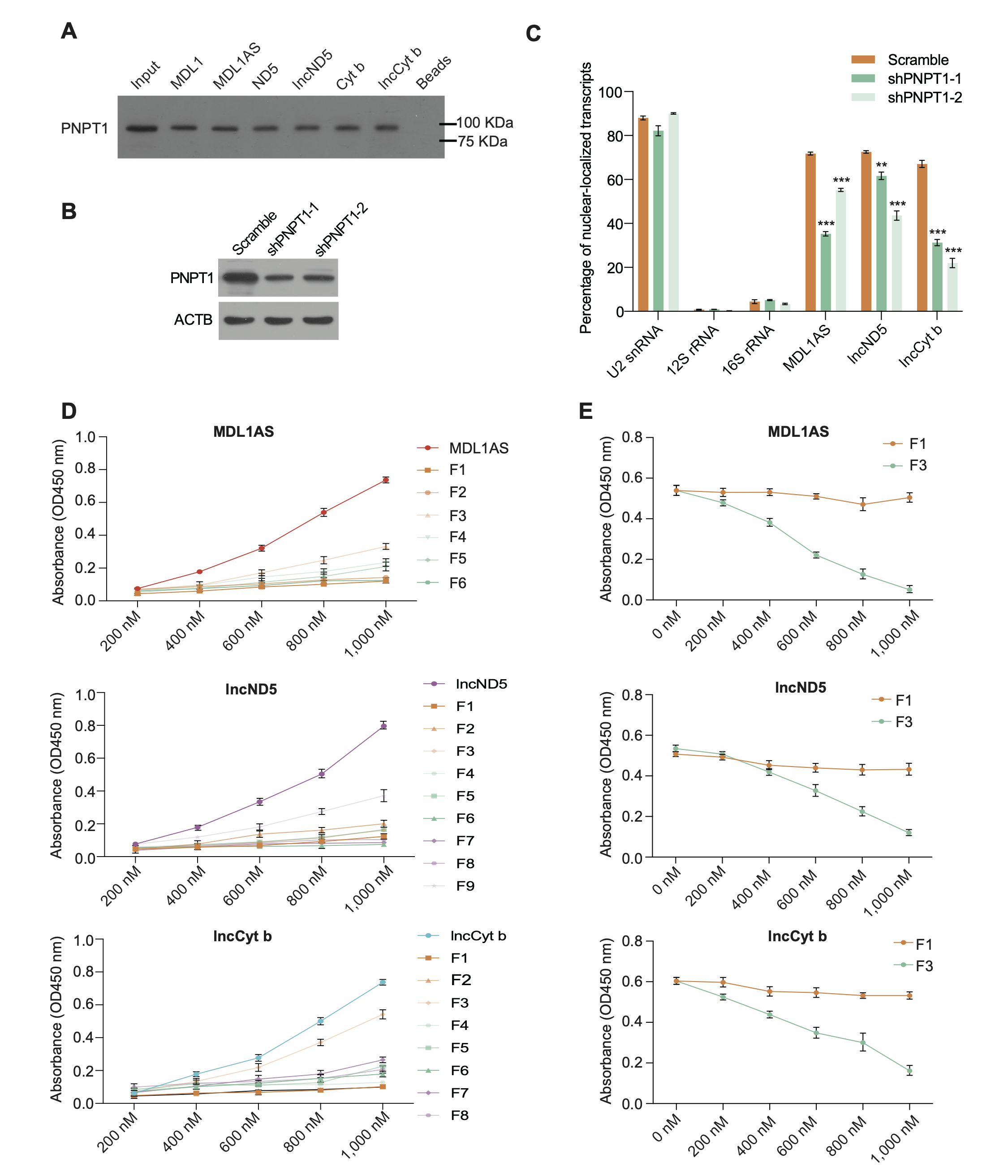

Supplement: S5 Fig — (A) Immunoblot detecting the PNPT1 level in the proteins pulled down by the indicated RNA baits. (B) Immunoblot detecting the shRNA-mediated PNPT1 knockdown in A549 cells. (C) Nuclear/cytoplasmic fractionation followed by RT-qPCR detecting the influence of PNPT1 knockdown on nuclear distribution of the indicated RNA transcripts in A549 cells. Data are shown as means ± SD of n = 3 independent experiments. **P < 0.01, ***P < 0.001 by Student’s t test. (D) ELISA assays detecting the association of PNPT1 protein with different regions of MDL1AS, lncND5 and lncCyt b. Data are shown as means ± SD of n = 3 independent experiments. (E) Competitive ELISA assays detecting effect of the lncCyt b segment F1 or F3 on PNPT1 association with MDL1AS, lncND5 and lncCyt b. Data are shown as means ± SD of n = 3 independent experiments. (TIFF) [file pgen.1011580.s005.tiff]

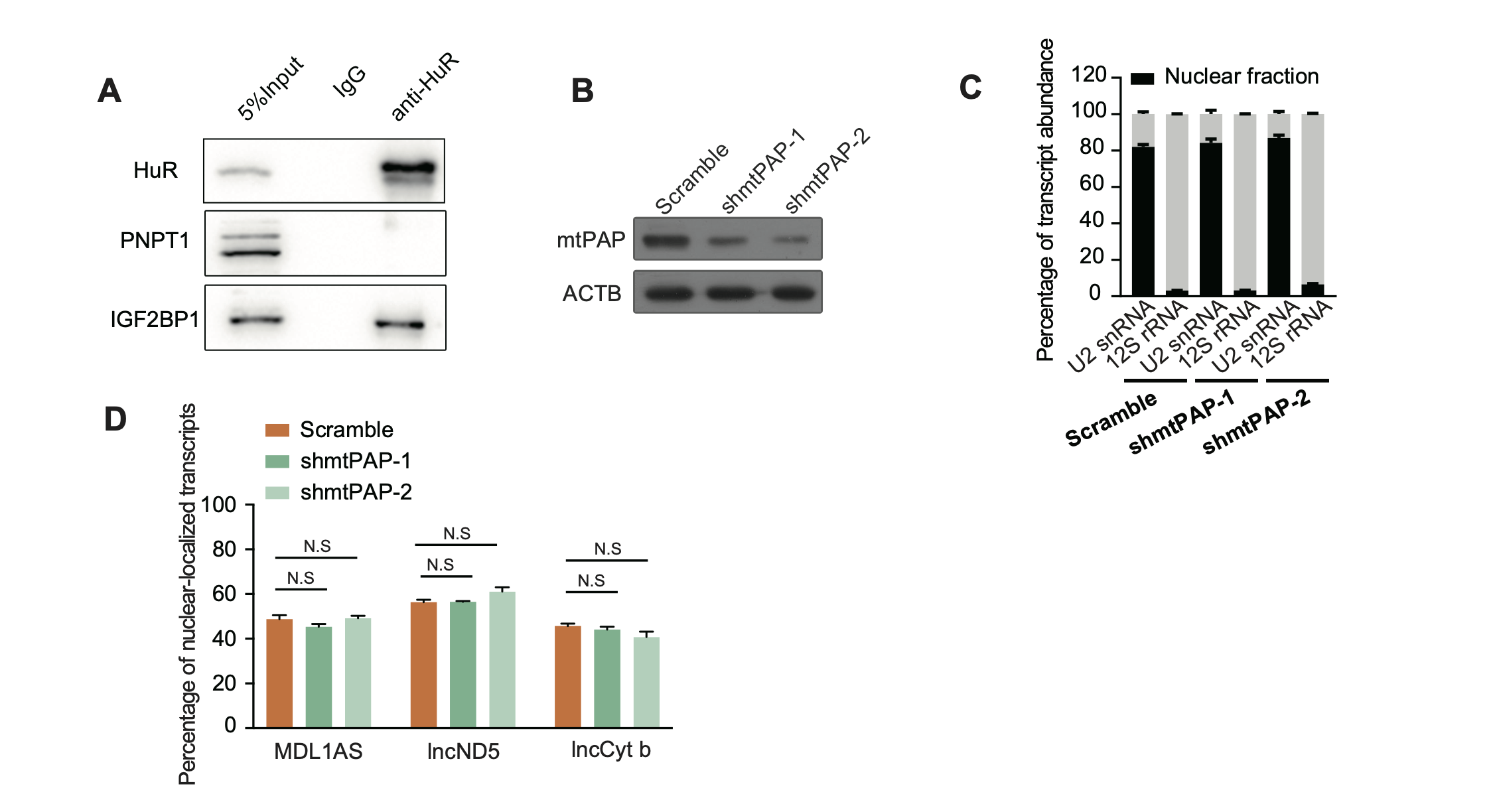

Supplement: S6 Fig — (A) Co-IP assay with anti-HuR antibody detecting the HuR/PNPT1 interaction in whole-cell lysates of A549 cells. IGF2BP1 was included as a control of the HuR-binding partner. (B) Immunoblot detecting the shRNA-mediated mtPAP knockdown in A549 cells. (C) Nuclear/cytoplasmic fractionation followed by RT-qPCR detecting the distribution of U2 snRNA and 12S rRNAs to confirm the findings of our nuclear/cytoplasmic fractionation in (D). Data are shown as means ± SD of n = 3 independent experiments. (D) Nuclear/cytoplasmic fractionation followed by RT-qPCR detecting the influence of mtPAP knockdown on the nuclear distribution of MDL1AS, lncND5 and lncCyt b in A549 cells. Data are shown as means ± SD of n = 3 independent experiments. N.S, not significant. (TIFF) [file pgen.1011580.s006.tiff]

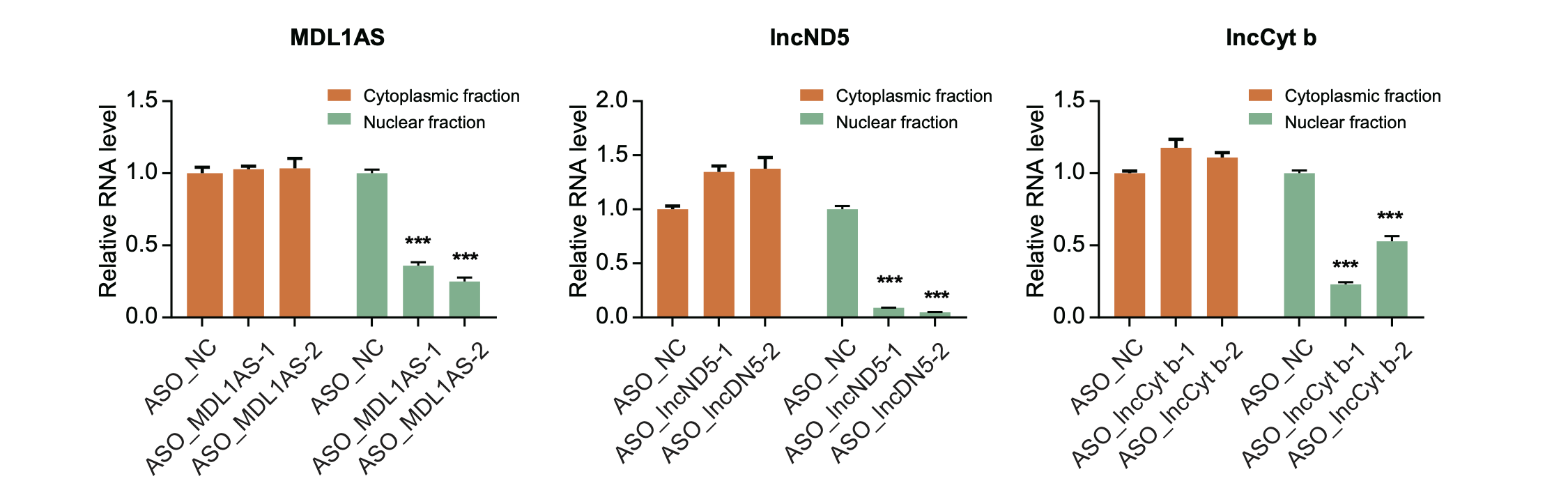

Supplement: S7 Fig — Data are shown as means ± SD of n = 3 independent experiments. ***P < 0.001 by Student’s t test. (TIFF) [file pgen.1011580.s007.tiff]

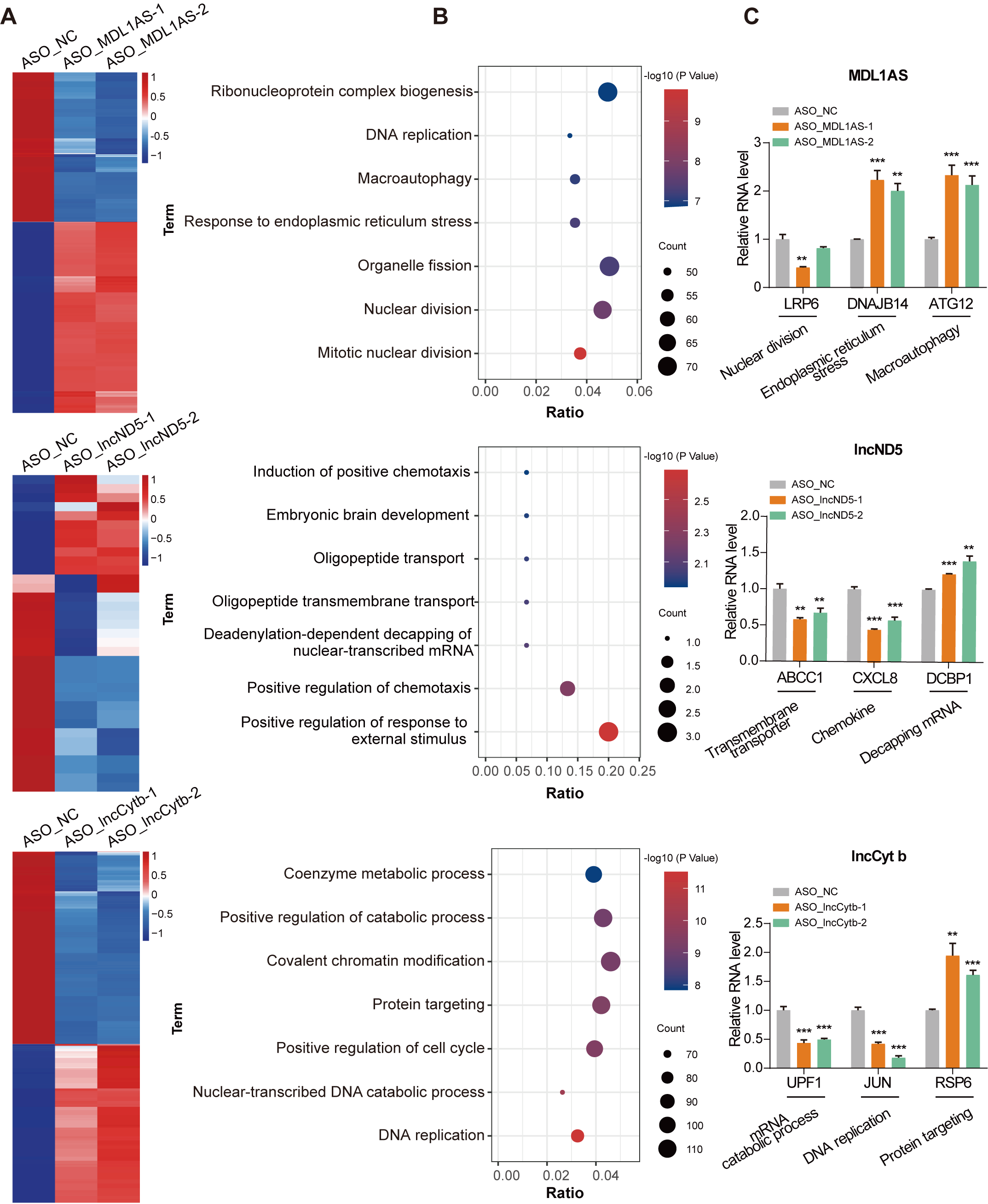

Supplement: S8 Fig — (A) Heatmaps showing the changes in nuclear gene expression compared with the negative control after knockdown of MDL1AS, lncND5 and lncCyt b with two independent ASOs. (B) GO enrichment analysis of the nuclear genes regulated by MDL1AS, lncND5 and lncCyt b. (C) RT-qPCR validating nuclear genes regulated by MDL1AS, lncND5 and lncCyt b. Specialized biological functions of the nuclear genes are annotated. Data are shown as means ± SD of n = 3 independent experiments. **P < 0.01, ***P < 0.001 by Student’s t test. (TIF) [file pgen.1011580.s008.tif]

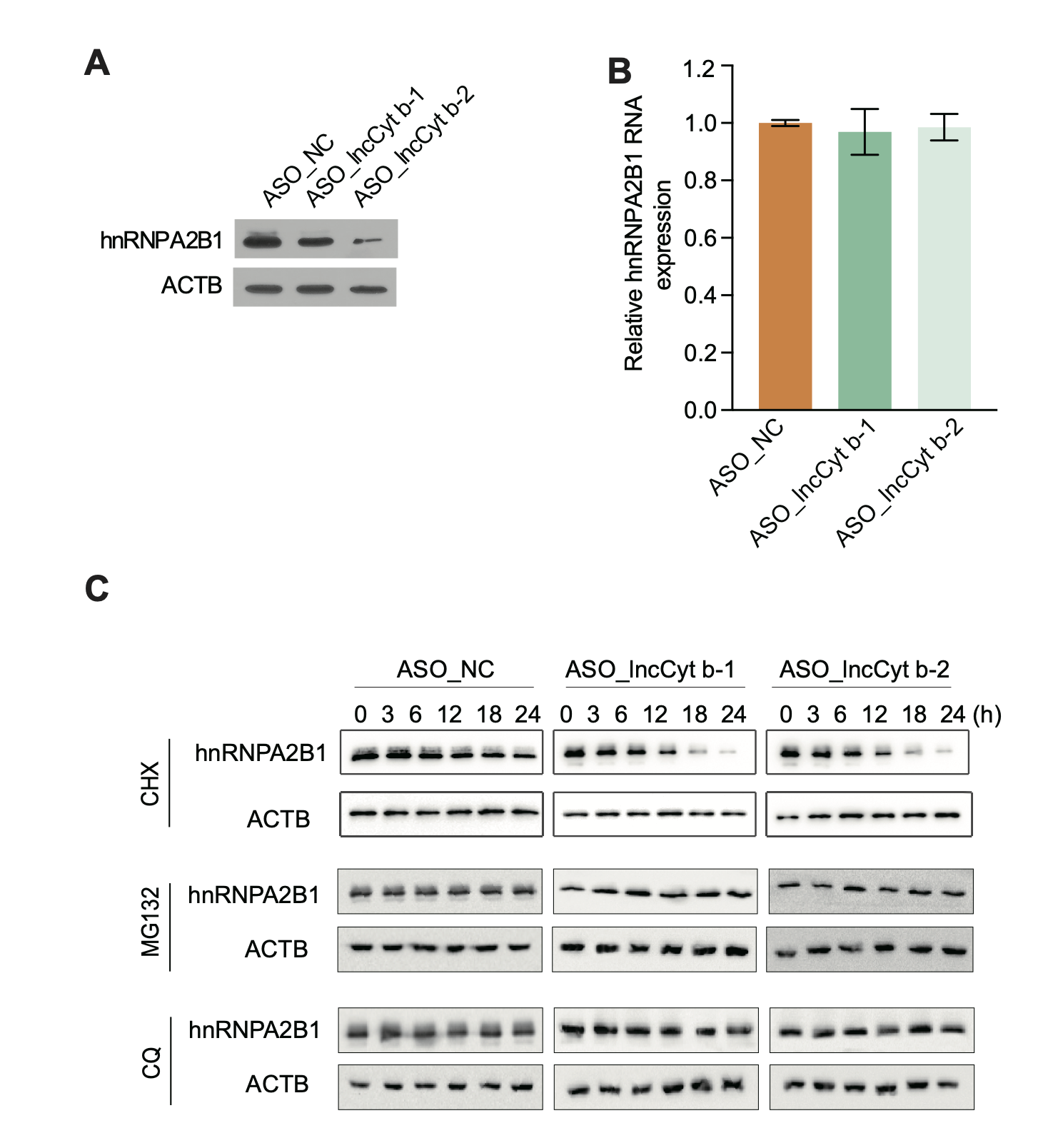

Supplement: S9 Fig — (A) Immunoblot detecting influence of lncCyt b knockdown on the hnRNPA2B1 protein level in A549 cells. (B) RT-qPCR detecting influence of lncCyt b knockdown on the hnRNPA2B1 mRNA level in A549 cells. (C) Immunoblot detecting the hnRNPA2B1 protein level in control and lncCyt b-depleted A549 cells after the treatment with CHX, MG132 or CQ at the indicated time points. (TIFF) [file pgen.1011580.s009.tiff]

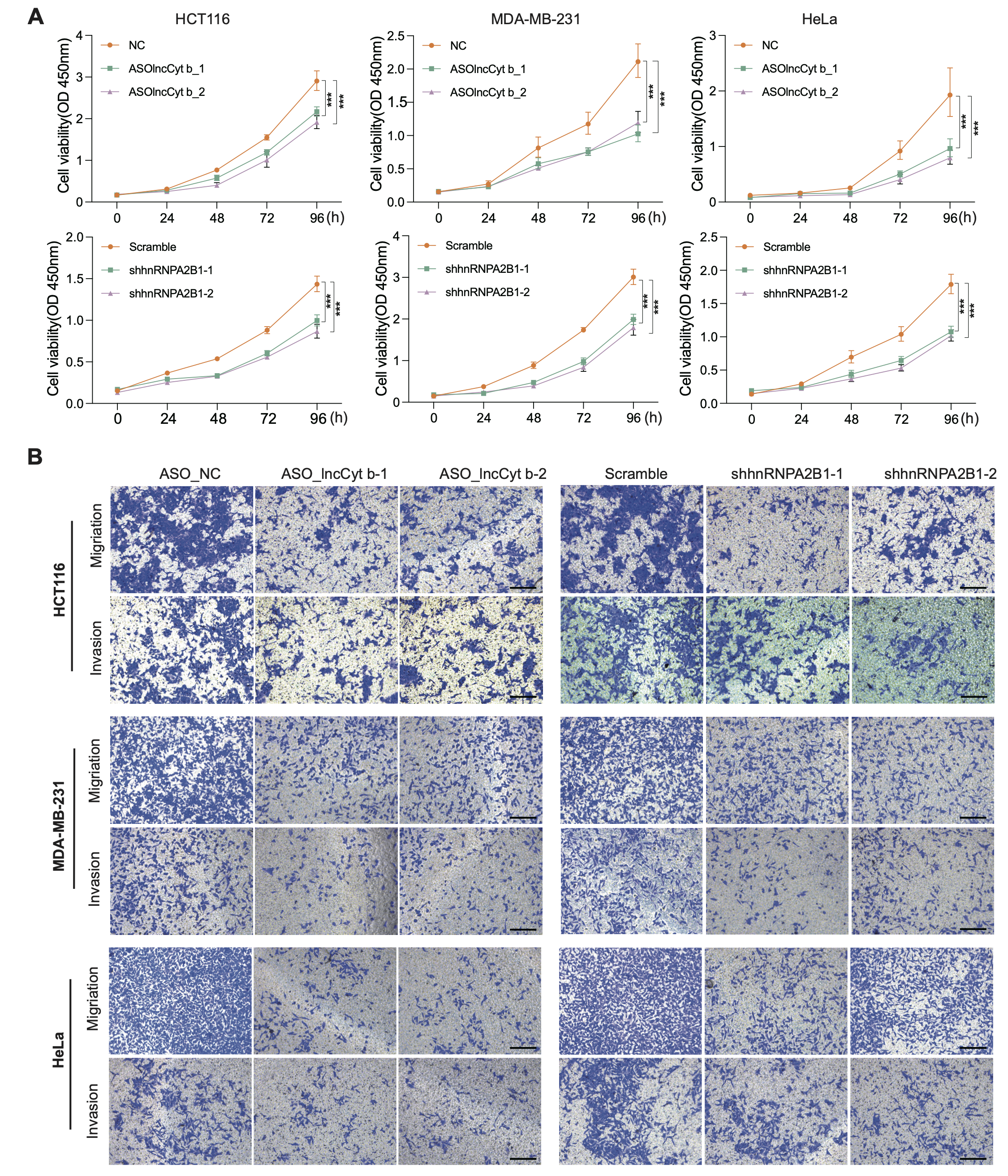

Supplement: S10 Fig — (A) CCK-8 assays showing the deceased proliferation of HCT116, MDA-MB-231 and HeLa cells by lncCyt b or hnRNPA2B1 knockdown. Data are shown as means ± SD of n = 3 independent experiments. ***P < 0.001 by Student’s t test. (B) Transwell assays showing the weakened migration and invasion of HCT116, MDA-MB-231 and HeLa cells by lncCyt b or hnRNPA2B1 knockdown. Scale bars, 100 μm. (TIFF) [file pgen.1011580.s010.tiff]

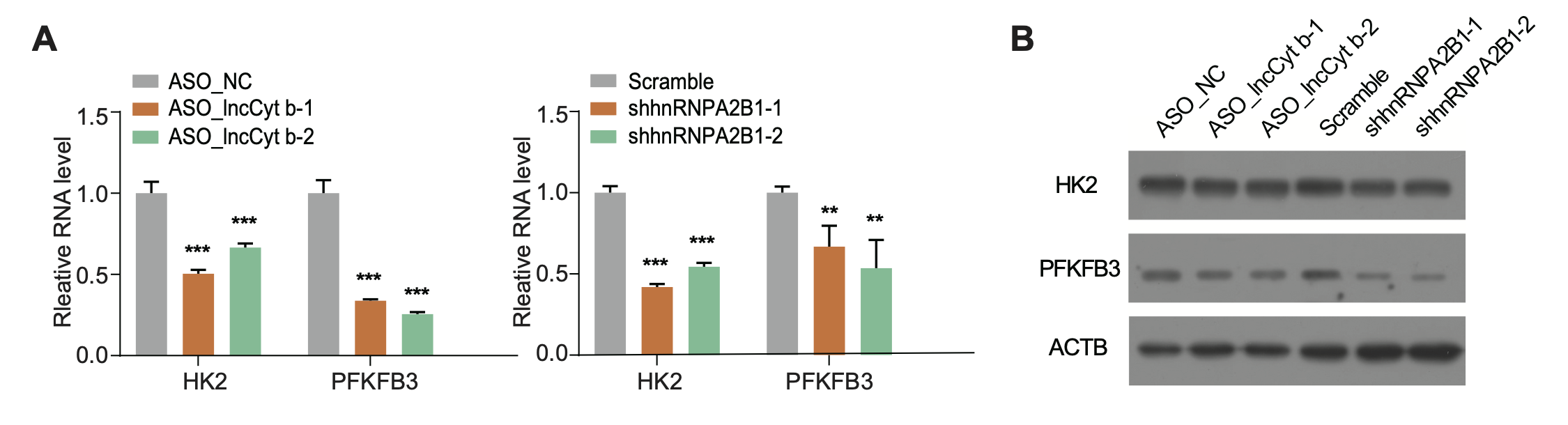

Supplement: S11 Fig — (A) RT-qPCR detecting influence of lncCyt b or hnRNPA2B1 knockdown on the RNA level of the indicated glycolysis-related genes. Data are shown as means ± SD of n = 3 independent experiments. **P < 0.01, ***P < 0.001 by Student’s t test. (B) Immunoblot detecting influence of lncCyt b or hnRNPA2B1 knockdown on the protein level of the indicated glycolysis-related genes. (TIFF) [file pgen.1011580.s011.tiff]
